# Dual-Functional Photonic Metacoating Integrating Fluorescence Thermometry and High-Performance Space Radiative Cooling

**DOI:** 10.1007/s40820-026-02195-8

**Published:** 2026-04-29

**Authors:** Hao Gong, Zhongyang Wang, Yan Zheng, Liping Tong, Hongchao Li, Zhiyuan Zhao, Junjia Liu, Gang Liu, Xiao Zhou, Tongxiang Fan

**Affiliations:** 1 State Key Laboratory of Metal Matrix Composites, School of Materials Science and Engineering, Shanghai Jiao Tong University, Shanghai, 200240 People’s Republic of China; 2 School of Materials, Shanghai Dianji University, Shanghai, 201306 People’s Republic of China; 3 Shanghai Institute of Spacecraft Equipment, Shanghai, 200240 People’s Republic of China

**Keywords:** Photonic metacoating, Eu-doped ZrO_2_ submicrosphere, Space radiative cooling, Fluorescence thermometry

## Abstract

Fluorescence thermometry offers a non-contact strategy for early detection of thermal instabilities on complex spacecraft surfaces, enabling reliable in-orbit temperature mapping. However, simultaneously achieving high-sensitivity fluorescence thermometry and efficient space radiative cooling remains challenging, as enhanced visible absorption improves thermometric response but increases solar heating. Here, we address this trade-off through a material-structure co-design strategy by developing an Eu-doped ZrO_2_ submicrosphere metacoating that integrates space radiative cooling with fluorescence-based temperature sensing. Guided by photonic-structure optimization using a constrained-gradient optimizer combined with grid-search mapping, the optimized metacoating, featuring a submicrosphere diameter of 0.756 µm and a volume fraction of 35%, achieves an ultralow solar absorptance (*α*_*s*_ = 0.076) and a high thermal emittance (*ε* = 0.931). In parallel, bandgap-driven compositional optimization identifies an optimal Eu content of 8.48%, enabling outstanding thermometric performance. The metacoating delivers a net cooling power of 323.69 W m^−2^ and a 77 °C temperature reduction relative to an Al sheet, outperforming representative oxide-based inorganic coatings. It allows temperature sensing over 173–433 K with a maximum relative sensitivity of 0.797% K^−1^, surpassing fluorescent oxides with comparable absorption edges. Moreover, the metacoating maintains the lowest *α*_*s*_ and reliable irradiation resistance under proton, electron, atomic oxygen and ultraviolet exposures, outperforming reported counterparts. Together with its scalable fabrication, this work establishes a dual-functional metacoating platform for intelligent spacecraft thermal management that combines efficient radiative cooling with high-sensitivity fluorescence thermometry.

**Supplementary Information:**

The online version contains supplementary material available at 10.1007/s40820-026-02195-8.

## Highlights

- Dual-functional Eu-doped ZrO_2_ submicrosphere metacoating for space radiative cooling and fluorescence thermometry.
- Photonic structure and materials co-design yield an ultralow solar absorptance (*α*_*s*_ = 0.076) and high emittance (*ε* = 0.931) in a ~ 100 μm thick metacoating.
- The metacoating achieves a net cooling power of 323.69 W m^−2^ and enables 173–433 K non-contact thermometry (*S*_*r,max*_ = 0.797% K^−1^) with reliable irradiation resistance under space environments.

**Supplementary Information:**

The online version contains supplementary material available at 10.1007/s40820-026-02195-8.

## Graphical abstract

**Figure:**
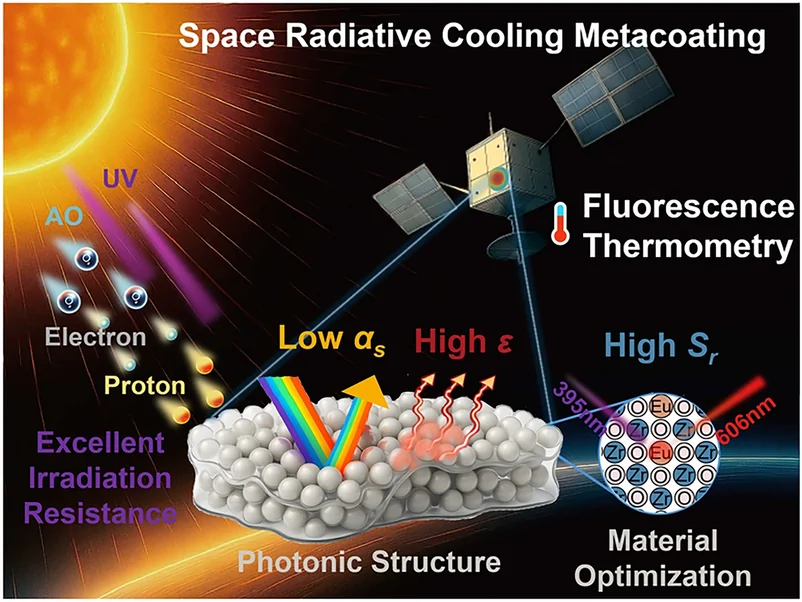

## Introduction

Thermal management defines the operational limits of spacecraft, as temperature fluctuations can directly compromise the performance of high-power missions [1]. Traditional radiative cooling coatings combining low solar absorptance (*α*_*s*_), high thermal emittance (*ε*) and thin-film design offer a transformative, zero-energy route to passive thermal regulation by efficiently dissipating heat to the deep-space cold sink [2–5]. To suppress solar input, high refractive index or wide bandgap (*E*_*g*_) oxides such as ZnO [6], Zn_2_SiO_4_ [7], MgGa_2_O_4_ [8] and ZrO_2_ [9], are typically employed to maximize solar backscattering. However, conventional oxide coatings lack in situ health monitoring capability, making the integration of precise and rapid temperature sensing essential for early detection of thermal anomalies and for maintaining spacecraft reliability. Traditional thermometric approaches (such as thermocouples, infrared thermography and thermosensitive paints) are hindered by wiring complexity, thermal perturbation or limited accuracy, rendering them unsuitable for extreme space environments [10, 11]. In contrast, fluorescence thermometry offers a non-contact and high-spatial-resolution optical solution that is independent of surface emissivity and resistant to electromagnetic interference, suiting the harsh space environment [12, 13]. Integrating such fluorescent thermometry into radiative cooling coatings thus represents a significant step toward intelligent thermal management systems that combine efficient passive cooling with real-time, self-perceptive temperature sensing.

Building on the concept of integrating fluorescence thermometry into radiative cooling coatings, the material must meet stringent requirements, combining a luminescent center that delivers strong and temperature-sensitive emissions with a host matrix that exhibits minimal *α*_*s*_ to maintain efficient radiative cooling performance. Rare-earth ions are ideal emitters because their 4*f* orbitals are shielded by outer 5*s* and 5*p* electrons, which suppresses environmental perturbations and yields sharp, well-defined emission lines [14, 15]. Parity-allowed 4*f*-5*d* transitions offer efficient excitation but introduce broadband visible absorption that can elevate *α*_*s*_, whereas parity-forbidden 4*f*-4*f* transitions are largely host-insensitive and arise from highly localized 4*f* states. These discrete intra-4*f* levels enable radiative processes without materially altering the host *E*_*g*_. Recent strategies have often employed narrow *E*_*g*_ host materials such as ZrTiO_4_ [16], Sr_2_NaMg_2_V_3_O_12_ [17], Ba_2_LuNbO_6_ [18] and LiY_6_(BO_3_)_3_O_5_ [19] to enhance thermometric sensitivity by strengthening the coupling between host absorption and rare-earth excitation or emission processes. However, this approach unavoidably increases visible absorption and degrades radiative cooling performance. A viable path is therefore to pair luminescent rare-earth ions with a wide *E*_*g*_ host that preserves low *α*_*s*_ while maintaining robust, temperature-responsive luminescence.

Overcoming the above trade-off between high-sensitivity fluorescence thermometry and minimal *α*_*s*_ remains a major challenge. To address this, Eu^3+^ ions offer a particularly promising route, as their excitation bands lie in the UV region and their large Stokes shift prevents self-absorption within the solar spectrum, thereby avoiding parasitic heating. The hypersensitive ^5^D_0_ → ^7^F_2_ transition in the red region shows narrow linewidths and strong temperature dependence, enabling reliable fluorescence-intensity-ratio (FIR) based thermometry [20, 21]. ZrO_2_ serves as an ideal host with a wide *E*_*g*_ and low phonon energy, which suppresses nonradiative losses and enhances emission efficiency [22, 23]. In addition, its high refractive index, combined with optimized submicrosphere-based random scattering photonic structure, facilitates maximized solar backscattering, thereby achieving ultralow *α*_*s*_ [24–27].

**Fig. 1:**
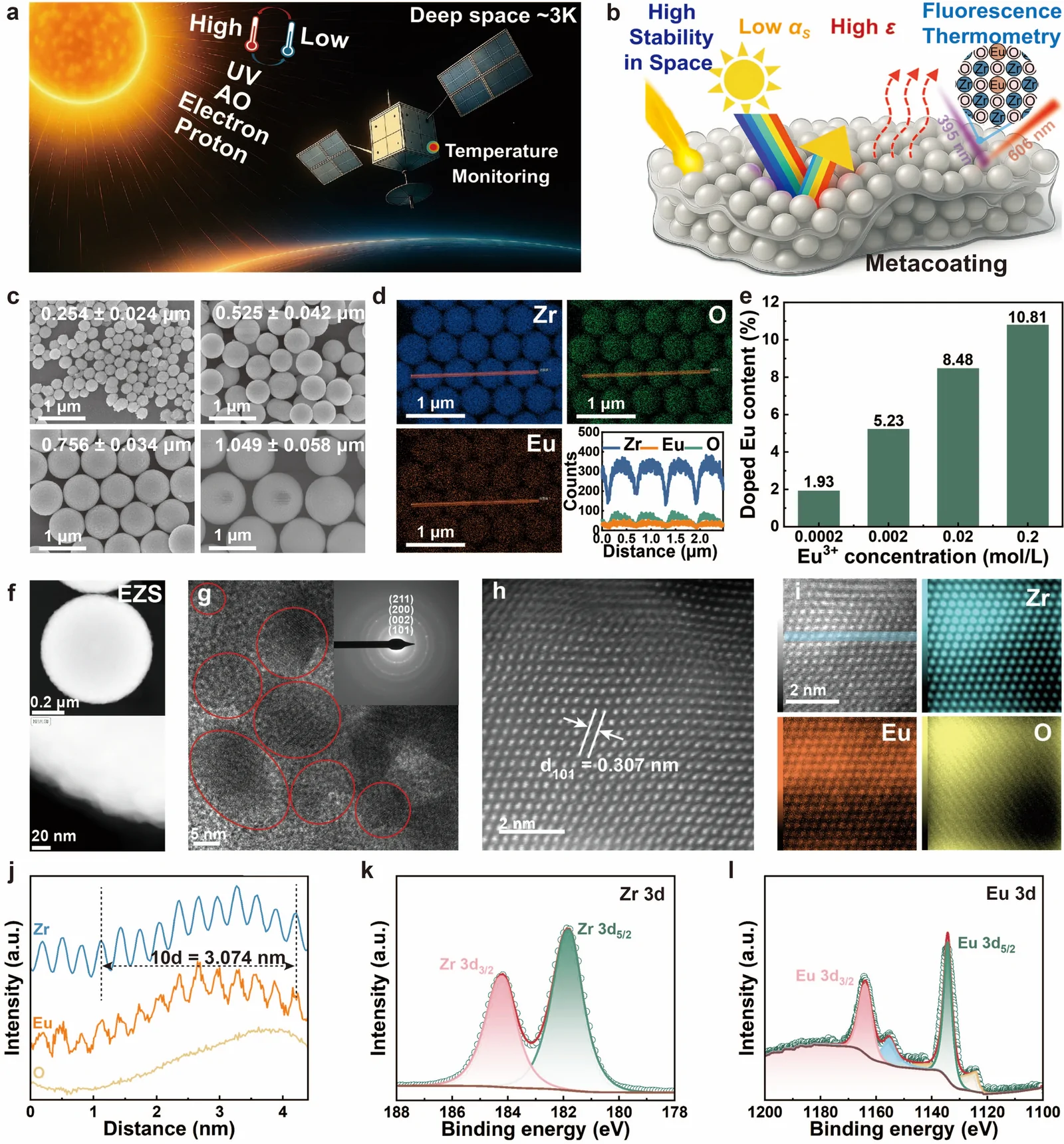
Working mechanism of the EZS metacoating and the characterization of EZS. **a** Schematic of proton, electron, AO and UV exposures, thermal cycling, and temperature monitoring on spacecraft surfaces. **b** Concept of the EZS metacoating integrating low *α*_*s*_, high *ε*, fluorescence thermometry and excellent durability under space environments. **c** SEM images of EZS with diameters of 0.254, 0.525, 0.756 and 1.049 μm. **d** EDS elemental mapping of 0.756 µm EZS. **e** Doped Eu content under different synthesis conditions. **f** HAADF-STEM images of an individual EZS and its rim region. **g** HR-TEM micrograph with the corresponding SAED pattern. **h** AC-HAADF-STEM microstructure and **i** atomic-scale EDS elemental mapping, **j** revealing the distribution of Zr, Eu and O atoms. **k** Zr 3*d* and **l** Eu 3*d* XPS spectra of EZS

Here, we present a dual-functional photonic metacoating that integrates high-sensitivity fluorescence thermometry with high-performance space radiative cooling, enabling real-time optical temperature readout while passively maintaining low surface temperatures under solar loading and stability under space irradiation environment, including proton, electron, atomic oxygen (AO), ultraviolet (UV), as well as thermal cycling (Fig. 1a, b). Guided by photonic structure design using a constrained gradient optimizer combined with grid search mapping, together with *E*_*g*_ engineering driven compositional optimization enabled by the controlled synthesis of Eu-doped ZrO_2_ submicrospheres (EZS), we identify an optimal Eu content of 8.48% and a photonic configuration with a submicrosphere diameter of 0.756 µm at a volume fraction of 35%. The resulting EZS metacoating delivers an ultralow *α*_*s*_ of 0.076 together with a high *ε* of 0.931, enabling a maximum net cooling power of 323.69 W m^−2^ and outperforming state-of-the-art counterparts. Under simulated vacuum space conditions, it reduces the temperature of an Al sheet by approximately 77 °C and achieves a significantly lower steady state temperature than conventional oxide coatings. Thermometrically, the metacoating attains a peak relative sensitivity *S*_*r*_ of 0.797% K^−1^ and exceeds systems with comparable or even shorter absorption edges (*λ*_*g*_ = 1240/*E*_*g*_), highlighting its superior thermometric sensitivity. The metacoating maintains consistently lowest *α*_*s*_ before and after proton, electron, AO, UV and combined irradiations, in contrast to the degradation observed in commercial and literature-reported coatings, underscoring its irradiation durability. Consequently, this work delivers the strong potential of an integrated, non-contact optical-thermometry and space radiative cooling metacoating for long-term, stable thermal management of spacecraft.

## Experimental Section

### Synthesis of EZS and Fabrication of Metacoating

EZS were prepared by modifying previously reported route for undoped ZrO_2_ submicrospheres (ZS) [28]. Typically, 0.4 mmol stearic acid, 20 mmol triethanolamine and a defined amount of water were dissolved in 200 mL of ethanol and stirred for 1 h. Zirconium propoxide solution (15 mmol) was then rapidly injected, and the mixture was left to react for a total of 150 min under gentle stirring. The resulting ZS precursor was collected by centrifugation and redispersed in 20 mL of ethanol. The particle size was tuned either by varying the water content (54, 40.5, or 27 mmol) or by replacing 25 mL of ethanol with methanol while keeping the water content at 36 mmol.

For Eu incorporation, the ZS precursor was transferred into 100 mL aqueous Eu^3+^ solutions (0.0002, 0.002, 0.02 or 0.2 mol L^−1^) in a 200 mL Teflon-lined autoclave and treated hydrothermally at 180 °C for 24 h. The resulting EZS precursors were separated by centrifugation, washed, dried at 120 °C for 4 h, and then calcined at 600, 800, 1000 or 1200 °C to induce crystallization. The final EZS exhibited mean diameters of 0.254, 0.525, 0.756 and 1.049 μm, respectively.

Metacoatings with EZS volume fractions of 15%, 25%, 35% and 45% were produced by spray coating. A diluted aqueous K_2_SiO_3_ binder solution was mixed with EZS and ball-milled at 120 rpm for 180 min to obtain a homogeneous slurry, which was then sprayed onto pre-sanded Al substrates. After drying at ambient conditions for 48 h, uniform metacoatings with various thicknesses were obtained. As a direct comparison to our previously reported coating [29], ZnO, TiO_2_ and Zn_2_TiO_4_ coatings were fabricated using the same K_2_SiO_3_ matrix and spray-coating protocol, with a fixed pigment volume fraction of 35% and a comparable thickness of ~ 100 μm.

### Optical Performance Characterizations

The optical *E*_*g*_ and reflectance were measured using a UV–Vis-NIR spectrophotometer (PerkinElmer Lambda 950, USA) equipped with a 15 cm integrating sphere. Spectra were collected from 0.25 to 2.5 μm, using polytetrafluoroethylene as the reflectance standard. Thermal emittance in the 2.5–25 μm range was obtained with a Fourier transform infrared spectrometer (FTIR, Bruker INVENIO-R, Germany) coupled to a gold-coated integrating sphere (Bruker A562). Fluorescence properties were characterized by using a fluorescence spectrometer (FLS920, Edinburgh Instruments, UK). Excitation and emission spectra were further measured on a spectrofluorometer (Horiba Jobin Yvon Fluorolog-3, HORIBA Jobin Yvon, France) equipped with a 450 W Xenon lamp. The same system, fitted with an integrating sphere, was used to determine fluorescence lifetime decay.

### Field Tests

Radiative cooling performance was evaluated in a vacuum blackbody chamber maintained below 10^–3^ Pa. Samples were illuminated with an AM0 solar simulator (SSS-500-A, Nmerry Technology Co., Ltd., China). A polyimide heater (~ 2 W) attached to the rear of the coated Al substrate mimicked internal heat generation. Temperatures were monitored in real time using a CENTER 309 thermometer (Qunte Technology Co., Ltd., Taiwan), with the thermocouple routed through a vacuum feedthrough to ensure accurate measurements.

Irradiation resistance was examined at the Shanghai Institute of Spacecraft Equipment using dedicated radiation facilities. Tests were conducted at 298 ± 5 K and ~ 10^–5^ Pa. Proton irradiation was performed at 50 keV with a fluence of 1.0 × 10^12^ p cm^−2^, while electron irradiation used 50 keV electrons at 1.0 × 10^14^ e cm^−2^. UV exposure was applied using deuterium and xenon lamps with an acceleration factor of ~ 5, corresponding to 1000 equivalent solar hours. AO tests were carried out using a SimulTek CompactAO system (SimulTek Research Co., Ltd.) with an average AO energy of 5 eV and a total fluence of 1 × 10^20^ atoms cm^−2^.

### Density Functional Theory (DFT) Calculations

DFT calculations of Eu-doped ZrO_2_ were carried out using the Vienna Ab initio Simulation Package (VASP). Ground-state electronic structures and formation energies were first obtained with the Perdew-Burke-Ernzerhof (PBE) exchange–correlation functional within the generalized gradient approximation (GGA) and projected augmented-wave (PAW) pseudopotentials [30, 31]. Eu-doped ZrO_2_ was modeled using a supercell approach, with a plane-wave cutoff energy of 600 eV and a 2 × 2 × 3 Monkhorst–Pack *k*-point mesh for Brillouin-zone sampling. All structures were relaxed until the residual forces on each atom were below 5 × 10^–4^ eV atom^−1^. Finite-temperature electronic structures and formation energies at 300 K were evaluated using an electron–phonon coupling scheme [32]. Since GGA-PBE is known to underestimate bandgaps in systems containing correlated *d* and *f* states, the *E*_*g*_ and electronic structure were further refined using the HSE06 hybrid exchange–correlation functional, which provides a more reliable description of the electronic self-energy [33].

### Numerical Simulation

The optical simulation was evaluated by combining Mie scattering theory and Monte Carlo (MC) ray tracing method [34–37]. For an isolated submicrosphere, the scattering (*Q*_sca_), extinction (*Q*_ext_) and absorption (*Q*_abs_) efficiencies were obtained from classical Mie scattering theory:

1 $$Q_{{{\mathrm{sca}}}} \left( {m, \chi } \right) = \frac{2}{{\chi^{2} }}\mathop \sum \limits_{n = 1}^{\infty } \left( {2n + 1} \right)\left( {\left| {a_{n} } \right|^{2} + \left| {b_{n} } \right|^{2} } \right)$$

2 $$Q_{{{\mathrm{ext}}}} \left( {m, \chi } \right) = \frac{2}{{\chi^{2} }}\mathop \sum \limits_{n = 1}^{\infty } \left( {2n + 1} \right){\mathrm{Re}} \left\{ {a_{n} + b_{n} } \right\}$$

3 $$Q_{{{\mathrm{abs}}}} = Q_{{{\mathrm{ext}}}} \left( {m, \chi } \right) - Q_{{{\mathrm{sca}}}} \left( {m, \chi } \right)$$

The Mie coefficients of *a*_*n*_ and *b*_*n*_ are given by:

4 $$a_{n} = \frac{{m\psi _{n} \left( {m\chi } \right)\psi ^{\prime}_{n} \left( \chi \right) - \psi _{n} \left( \chi \right)\psi ^{\prime}_{n} \left( {m\chi } \right)}}{{m\psi _{n} \left( {m\chi } \right)\xi ^{\prime}_{n} \left( \chi \right) - \psi ^{\prime}_{n} \left( {m\chi } \right)\xi _{n} \left( \chi \right)}}$$

5 $$b_{n} = \frac{{\psi _{n} \left( {m\chi } \right)\psi ^{\prime}_{n} \left( \chi \right) - m\psi _{n} \left( \chi \right)\psi ^{\prime}_{n} \left( {m\chi } \right)}}{{\psi _{n} \left( {m\chi } \right)\xi ^{\prime}_{n} \left( \chi \right) - m\xi _{n} \left( \chi \right)\psi ^{\prime}_{n} \left( {m\chi } \right)}}$$

where ${\psi }_{n}$ and ${\xi }_{n}$ are Riccati-Bessel functions; $\chi = \frac{\pi D}{\lambda }$ is the size parameter; $m=\frac{n+ik}{{n}_{0}}$ is the complex refractive index normalized to the surrounding medium.

For metacoatings composed of EZS submicrospheres embedded in a K_2_SiO_3_ binder, assuming independent scattering, the scattering coefficient (${k}_{\mathrm{sca}}$) and anisotropy factor (g) were obtained by [35]:

6 $$k_{{{\mathrm{sca}}}} = f_{v} \int\limits_{{D_{1} }}^{{D_{n} }} {k_{{{\mathrm{sca}}}} \left( D \right)\frac{{\pi D^{3} }}{6}n\left( D \right){\mathrm{d}}D = \frac{{3f_{v} }}{2}} \int\limits_{{D_{1} }}^{{D_{n} }} {Q_{{sca}} \left( D \right) \cdot \frac{{\pi D^{2} }}{6}n\left( D \right){\mathrm{d}}D}$$

7 $$g = \int\limits_{{D_{1} }}^{{D_{n} }} {\frac{{k_{{{\mathrm{sca}}}} \left( D \right)}}{{k_{{{\mathrm{sca}}}} }}~\mathop \smallint \limits_{0}^{{2\pi }} \Phi \left( {\theta ,D} \right)\sin \theta \cos \theta {\mathrm{d}}\theta ~\frac{{\pi D^{3} }}{6}n\left( D \right){\mathrm{d}}D = } \int\limits_{{D_{1} }}^{{D_{n} }} {\frac{{k_{{{\mathrm{sca}}}} \left( D \right)}}{{k_{{{\mathrm{sca}}}} }}~g\left( D \right)\frac{{\pi D^{3} }}{6}n\left( D \right){\mathrm{d}}D}$$

where $\mathrm{n}\left(D\right)\mathrm{d}D$ is the probability density function between D and $D+\mathrm{d}D$. The diameter distribution follows a log-normal form [38, 39]:

8 $$n\left( D \right) = \frac{1}{{D\sigma \sqrt {2\pi } }}\exp \left( { - \frac{{\left( {\ln D - \mu } \right)^{2} }}{{2\sigma^{2} }}} \right)$$

where (μ) and (σ) are the geometric mean diameter and geometric standard deviation, respectively.

The total backscattering strength (${S}_{t}$) of a coating with thickness (H) was written as:

9 $$S_{t} = S_{{{\mathrm{sca}}}} \cdot H = k_{{{\mathrm{sca}}}} \cdot H \cdot \left( {1 - g} \right)/2$$

At higher volume fractions, dependent scattering effects were included via a correction factor (γ), giving:

10 $$S_{t}^{{{\mathrm{Dep}}}} = \gamma \cdot S_{t}$$

The effective radiative properties (${k}_{\mathrm{sca}}$, g, ${S}_{\mathrm{t}}^{\mathrm{Dep}}$) were then used as inputs for MC simulations of reflectance and transmittance. For each parameter set, 10^6^ photons were launched at normal incidence onto a layer of prescribed thickness. Photon paths were traced through the medium by stochastically sampling free paths, scattering angles (from the phase function), and absorption events based on the extinction coefficient. Photons exiting the entrance surface were counted as reflected, whereas those leaving the back surface were counted as transmitted. The spectral reflectance *R*(*λ*) and transmittance *T*(*λ*) were obtained by normalizing reflected and transmitted weights to the incident total. To efficiently explore design space and identify low-*α*_s_ structures, the MC solver was coupled to two optimization strategies: a constrained-gradient routine for local optimization and a grid-search mapper for global scans over submicrosphere diameter, volume fraction and metacoating thickness [25].

## Results and Discussion

### Controlled Synthesis, Characterization and Modulation Fluorescence of EZS

Advancing the development of photonic metacoatings that integrate high-sensitivity fluorescence thermometry with high-performance space radiative cooling, we developed a controlled synthesis of EZS guided by our previously established oligomeric aggregation pathway [28]. Forced hydrolysis of alkoxide oligomeric precursors with concurrent Eu^3+^ coordination under hydrothermal conditions enabled precise control over both submicropshere diameter and dopant loading (Figs. S1 and S2 ). Diameters were tuned to 0.254, 0.525, 0.756 and 1.049 µm under the optimized calcination, as validated by scanning electron microscopy (SEM) (Figs. 1c and S3–S6). The selected diameters cover the principal solar wavelengths for Mie scattering, allowing identification of the minimum-*α*_*s*_ configuration of metacoating for enhanced radiative cooling. The uniformly elemental distributions of Zr, O and Eu across submicrospheres with different diameters are confirmed by energy-dispersive X-ray spectroscopy (EDS) analysis, ensuring compositionally homogeneous building blocks for constructing the photonic metacoating (Figs. 1d and S7). In parallel, Eu contents were adjusted to 1.93%, 5.23%, 8.48% and 10.81%, as verified by inductively coupled plasma optical emission spectrometry (ICP-OES), providing a compositional space to optimize fluorescence thermometric performance (Fig. 1e).

Comprehensive structural and chemical characterizations were performed on the EZS. The EZS exhibit well-defined spherical morphology composed of nanocrystallites typically smaller than 10 nm (Figs. 1f, g). Selected-area electron diffraction (SAED) confirms a polycrystalline tetragonal phase, indicated by rings corresponding to the (101), (002), (200), and (202) planes (Fig. 1g) [40]. Rietveld refinement analysis indicates that Eu is incorporated into the Zr lattice sites within the P4_2_/nmc crystal structure (Fig. S8). Aberration-corrected high-angle annular dark-field scanning transmission electron microscopy (AC-HAADF-STEM) imaging shows lattice fringes of 0.307 nm, matching the (101) interplanar spacing of tetragonal ZrO_2_, while atomic-scale EDS mapping verifies selective substitution of Eu^3+^ at Zr sites (Fig. 1i) [41]. Although the O distribution could not be precisely mapped due to signal overlap, the periodic Zr/Eu arrangement spanning ten atomic sites shows a length of 3.074 nm, corresponding to approximately ten times the (101) lattice spacing, which confirms the homogeneous Eu incorporation (Fig. 1j). X-ray photoelectron spectroscopy (XPS) analysis further confirms the chemical states of the constituent elements (Fig. S9). The Zr 3*d* spectrum shows a doublet at 181.79 and 184.18 eV (Δ*E* = 2.39 eV), consistent with the presence of Zr^4+^ in the lattice (Fig. 1k) [42, 43]. The Eu 3*d* spectrum displays a typical doublet at 1134.28 eV (Eu 3*d*_5/2_) and 1163.90 eV (Eu 3*d*_3/2_) with a splitting of 29.62 eV, confirming successful Eu^3+^ doping (Fig. 1l). Additional satellite peaks observed at 1124.11 and 1155.25 eV suggest enhanced 4*f* electron localization, plausibly arising from *V*_*O*_-mediated charge-transfer processes [44, 45].

**Fig. 2:**
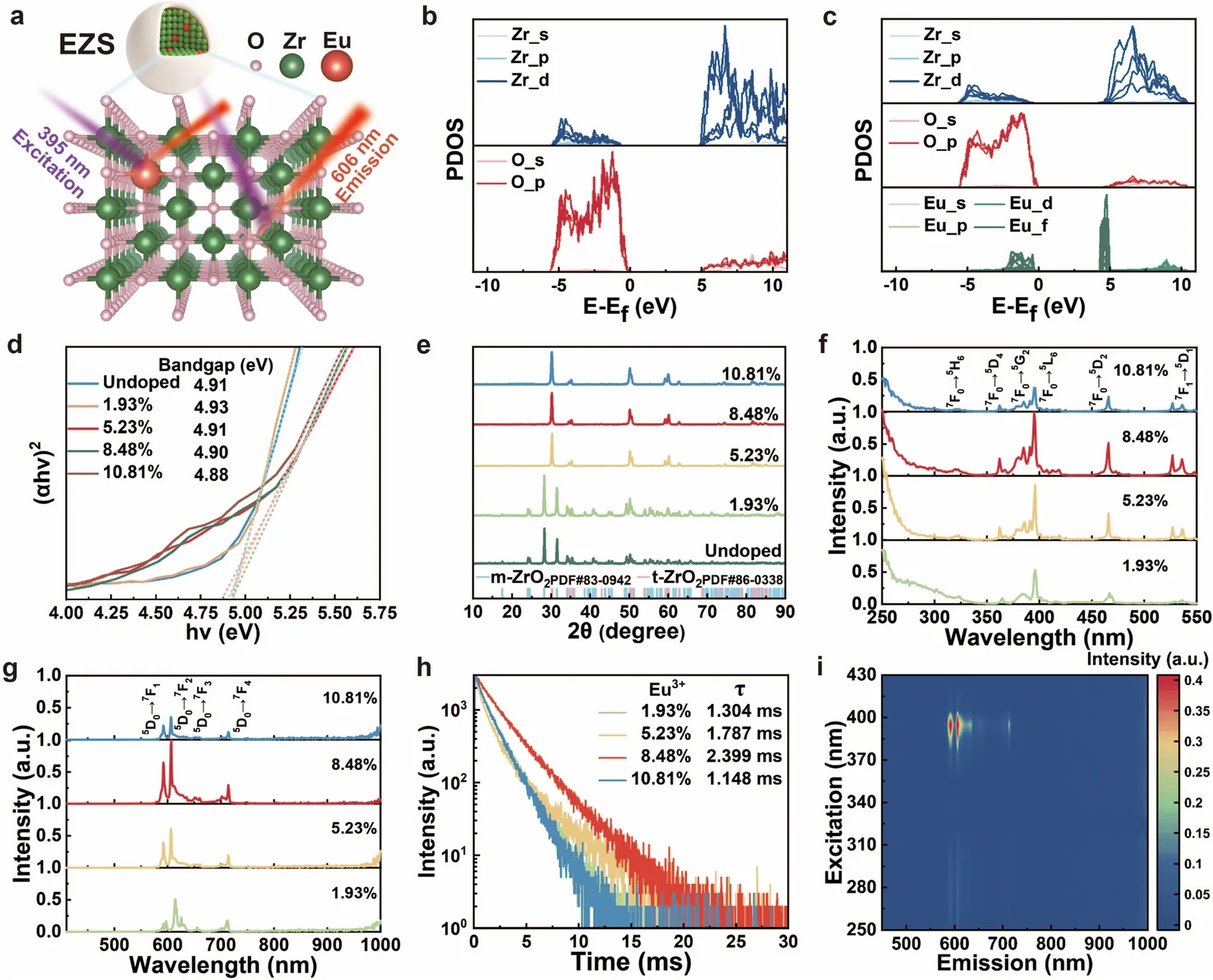
Modulation of EZS optical and photoluminescent properties. **a** Tetragonal crystal structure of EZS with Eu^3+^ as luminescent centers. PDOS of **b** undoped and **c** 8.33% Eu-doped ZrO_2_. **d** Optical *E*_*g*_ derived from Tauc plots, **e** XRD patterns, **f** excitation spectra, **g** emission spectra, **h** fluorescence decay curves (λ_em_ = 606 nm and λ_ex_ = 395 nm) at different doping concentrations and **i** excitation-emission contour map of 8.48% EZS

To enable non-contact fluorescence thermometry in EZS metacoatings for space radiative cooling, we systematically investigated the effects of Eu doping on the optical *E*_*g*_ and fluorescence behavior. DFT calculations were performed on Eu-doped or pristine ZrO_2_ (Figs. 2a–c and S10). In the projected density of states (PDOS) of pristine ZrO_2_, the valence band maximum (VBM) arises mainly from O 2*p* orbitals, while the conduction band minimum (CBM) originates from Zr 4*d* states [29, 46]. Upon Eu incorporation, the overall shape and position of both VB and CB remain largely unchanged, indicating that the intrinsic *E*_*g*_ of ZrO_2_ is essentially preserved. However, sharp and localized peaks of Eu^3+^ 4*f* states emerge near the CB edge. These 4*f* orbitals, effectively shielded by outer 5*s*^2^5*p*^6^ electrons, exhibit weak hybridization with the host lattice, confining excitation energy within Eu^3+^ ions and minimizing nonradiative loss to the matrix. The localized 4*f* states thus serve as efficient optical centers, governing both absorption and emission via 4*f*-4*f* transitions. This localized-state-mediated luminescence underpins efficient fluorescence thermometry by strengthening radiative transitions without largely perturbing the *E*_*g*_ and increasing *α*_*s*_.

Experimental optical characterizations further validate the theoretical findings. With increasing Eu doping under optimized calcination conditions, Tauc plot analysis of (*ahv*)^2^ versus photon energy (*hv*) reveals a slight reduction in the optical *E*_*g*_, consistent with DFT predictions (Figs. 2d and S11) [47]. This reduction arises primarily from the formation of *V*_*O*_ that accompanies Eu^3+^ substitution for Zr^4+^. Concurrently, Fourier transform infrared (FTIR) spectra show no significant changes in chemical composition (Fig. S12). X-ray diffraction (XRD) analysis reveals a structural transformation from the monoclinic phase (2*θ* = 24.1°, 28.2°, 31.5°, 34.1°, 50.2°; PDF#83-0942) to the tetragonal phase (2*θ* = 30.2°, 35.3°, 50.2°, 60.1°; PDF#86-0338) upon Eu doping at higher concentrations (Fig. 2e) [41]. Even at a high Eu content of 8.48%, the *E*_*g*_ remains nearly unchanged compared with undoped ZrO_2_, ensuring that the metacoating maintains its low *α*_*s*_.

We further investigated the fluorescence properties, and the excitation (*λ*_em_ = 606 nm) and emission (*λ*_ex_ = 395 nm) spectra are shown in Figs. 2f, g and S13. The broad band of 250–325 nm arises from O^2−^(2*p*) → Eu^3+^(4*f*) charge-transfer excitation, which is intense and sensitive to local coordination. Peaks at 320, 362, 385, 395, 465 and 527 nm correspond to the Eu^3+ 7^F_0_ → ^5^H_6_, ^7^F_0_ → ^5^D_4_, ^7^F_0_ → ^5^G_2_, ^7^F_0_ → ^5^L_6_, ^7^F_0_ → ^5^D_2_ and ^7^F_1_ → ^5^D_1_ transitions [23, 48]. Emission bands at 583–600, 600–637, 645–665 and 690–720 nm correspond to ^5^D_0_ → ^7^F_J_ (J = 1–4). The ^5^D_0_ → ^7^F_1_/^7^F_3_ bands are magnetic dipole, while ^5^D_0_ → ^7^F_2_/^7^F_4_ are electric dipole and intensify at non-centrosymmetric Eu^3+^ sites, with the splitting confirming complete lifting of the ^7^F_J_ degeneracy [49]. With increasing Eu content, both excitation and emission first increase and then decrease, giving an optimum at 8.48%. The decay curves show the lifetime follows the same trend, initially increasing with Eu content, reaching 2.399 ms at 8.48% and falling to 1.148 ms at 10.81% (Fig. 2h). The initial enhancement is attributed to two factors: more activator ions (Eu^3+^) per EZS directly increase emission centers and Eu substitution on Zr sites with charge-compensating *V*_*O*_ that break local centrosymmetry and promote energy transfer to Eu^3+^ [48]. At higher loading, over-strong charge-transfer absorption reduces the effective excitation of 4*f*-4*f* transitions, and concentration-quenching pathways such as cross-relaxation and defect-assisted nonradiative transfer dominate, reducing intensity and lifetime [49]. Undoped ZS show no detectable emission (Fig. S14), confirming that luminescence originates from Eu incorporation. The excitation-emission contour map reveals an active region spanning 385–400 nm in excitation and 580–640 nm in emission, predominantly governed by the ^7^F_0_ → ^5^L_6_, ^7^F_0_ → ^5^D_2_ and ^5^D_0_ → ^7^F_2_ transitions (Fig. 2i). Overall, 8.48% Eu content defines the optimal composition for yielding strong red emission and maximizing luminescent performance.

### Photonically Optimized Metacoatings for Minimized ***α***_***s***_ and Enhanced Radiative Cooling Performance

**Fig. 3:**
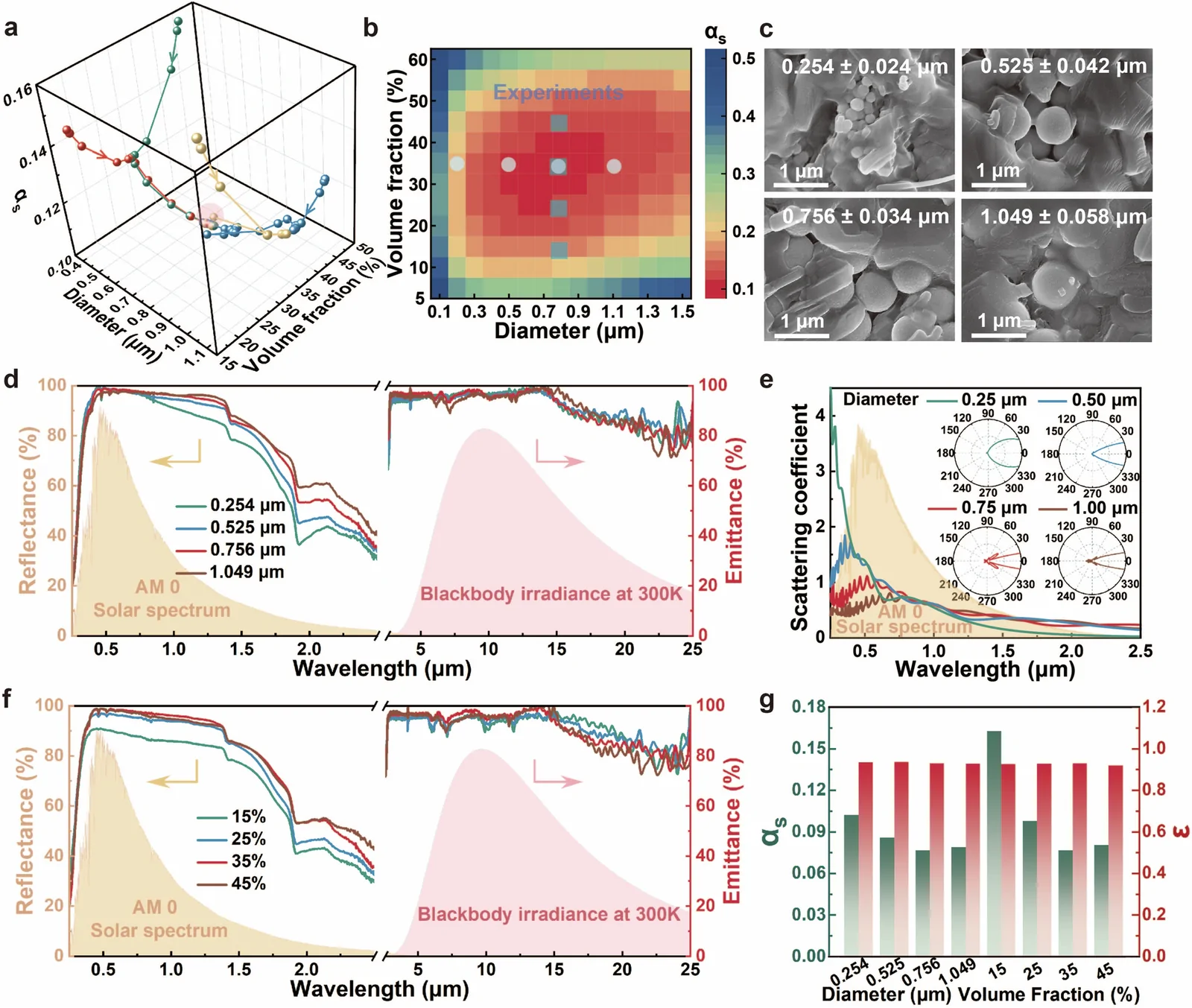
Photonic structure optimization of EZS metacoating. **a** Constrained gradient optimization and **b** grid search mapping of the optimal *α*_*s*_ region for 100 μm thick metacoatings as a function of diameter and volume fraction. **c** SEM images, **d** reflectance and emittance spectra of metacoatings with varying EZS diameters. **e** Scattering coefficient spectra of EZS with different diameters and corresponding phase functions at 0.5 µm for incident light (inset). **f** Reflectance and emittance spectra of EZS metacoatings with different volume fractions. **g** *α*_*s*_ and *ε* of EZS metacoatings with different diameters and volume fractions

Photonic structure optimization is pivotal for minimizing *α*_*s*_ and, in turn, maximizing radiative cooling performance. To support rational and fabrication-tolerant design, we combined a high-efficiency constrained-gradient optimization with a complementary grid-search map to chart the *α*_*s*_ landscape and delineate the optimal region across the EZS parameter space [25]. Using *α*_*s*_ minimization as the objective, the constrained-gradient routine converged to machine tolerance (e^−10^) and returned fast, accurate optima for the EZS diameter and volume fraction. As shown in Fig. 3a, multi-start optimizations launched from four contrasting combinations (small/large diameters × low/high volume fractions) collapse to a narrow basin at diameter of 0.70–0.75 µm and volume fraction of 32%–36%, identifying a global optimum for minimizing *α*_*s*_. To test global robustness and resolve response trends, we constructed a diameter-volume-fraction design grid and executed a full factorial sweep via grid search (Fig. 3b). For experimental validation, EZS metacoatings were fabricated using four representative diameters (0.254, 0.525, 0.766 and 1.049 µm) and volume fractions (15%, 25%, 35% and 45%). K_2_SiO_3_ was selected as the binder because its excellent optical transparency, strong irradiation resistance, water-based processability, and ability to form a robust Si–O–Si network provide key advantages for constructing stable photonic metacoatings [9]. SEM images show that the EZS are uniformly embedded and randomly dispersed within the K_2_SiO_3_ binder, forming a heterogeneous particulate architecture. Increasing the EZS diameter decreases the particle number density and produces a more sparsely distributed network of larger scattering centers (Figs. 3c and S15). Increasing the volume fraction from 15% to 45% progressively increases the packing density and interparticle connectivity, generating distinct photonic architectures (Fig. S16). Together, these parameters govern the size, density and spatial distribution of scattering centers within the metacoating, enabling comparative optical characterization.

Reflectance and emittance spectra under different structural parameters were conducted to evaluate optical performance. Reflectance varies with wavelength and diameter, with 0.254 µm EZS metacoating showing the highest reflectance in 0.38–0.52 µm, 0.756 µm EZS metacoating performing best in 0.52–0.87 µm, and reflectance increasing monotonically with diameter in 0.87–2.5 µm. These trends match the simulated profiles and arise from a red-shift of the scattering-efficiency maximum with increasing diameter, which enhances backscattering at longer wavelengths (Figs. 3e and S17). Phase-function analysis further confirms stronger backscattering for larger submicrospheres. Consistently, increasing the EZS diameter from 0.254 to 0.756 µm lowers *α*_*s*_ from 0.102 to 0.076, while a further increase to 1.049 µm yields a slight rebound to 0.079. *ε* shows only minor variation, remaining within 0.928–0.936 owing to the strong phonon polariton modes of K_2_SiO_3_ and EZS (Fig. 3g) [50, 51].

Volume fraction likewise governs the optical response. At volume fraction of 15%, the sparse scattering centers result in low broadband reflectance (Figs. 3f and S16). Raising the volume fraction to 35% increases backscattering and raises reflectance across the solar spectrum. At 45%, excessive particle crowding reduces effective scattering interfaces and depresses reflectance in the 0.54–1.37 µm region, in agreement with simulated results (Figs. 3f and S17). Accordingly, *α*_*s*_ drops from 0.163 to 0.076 as the volume fraction increases from 15% to 35%, while *ε* rises from 0.926 to 0.931. When the fraction reaches 45%, *α*_*s*_ slightly increases to 0.081 and *ε* declines to 0.920, underscoring that excessive loading compromises performance (Fig. 3g). As the thickness increases, the *α*_*s*_ first decreases and then remains nearly constant with slight fluctuations (Fig. S18). Specifically, increasing the metacoating thickness from 50 to 100 µm reduces *α*_*s*_ from 0.106 to 0.076 due to enhanced photon backscattering. Further thickening leads to only marginal changes, with *α*_*s*_ reaching 0.075 at 200 µm and slightly increasing to 0.077 at 300 µm due to increased near-infrared absorption in the K_2_SiO_3_ binder. Meanwhile, the *ε* gradually increases from 0.914 to 0.945 with thickness. Since further thickening beyond 100 µm provides limited optical benefits while increasing metacoating weight and cracking risk, a thickness of approximately 100 µm was selected as a practical optimum. In summary, for a ~ 100 µm-thick metacoating, an EZS diameter of 0.756 µm and a volume fraction of 35% yield a minimum *α*_*s*_ of 0.076 while maintaining a high *ε* of 0.931.

**Fig. 4:**
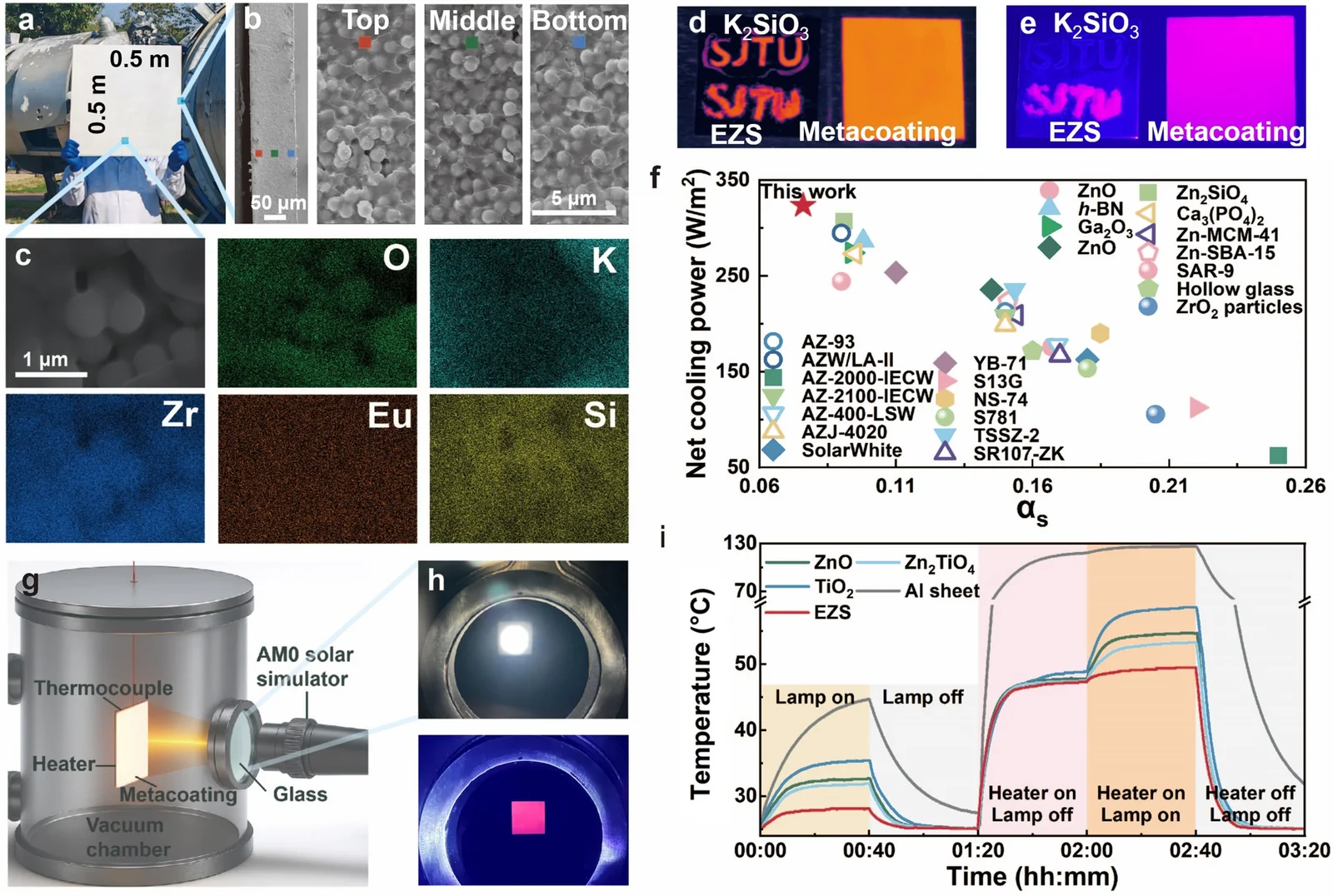
Structural, optical and radiative cooling characterization of the EZS metacoating. **a** Photograph (0.5 m × 0.5 m), **b** cross-sectional and **c** surface SEM images of EZS metacoating with corresponding EDS elemental mapping, showing about 100 μm thickness and homogeneous EZS dispersion. **d** Infrared thermal image after thermal equilibrium at 50 °C and **e** optical photograph under 395 nm excitation for EZS, K_2_SiO_3_ and the metacoating on an Al sheet. **f** Comparison of net cooling power between the EZS metacoating and other all-inorganic radiative cooling coatings across varying *α*_*s*_. **g** Schematic of the vacuum experimental setup simulating space conditions. **h** EZS metacoating under AM0 illumination and 395 nm excitation. **i** Temperature evolution of the EZS metacoating, Al sheet, and reference coatings during lamp on–off cycles, with and without heater input

The metacoating can be readily scaled up and fabricated using an industrially compatible spray process, producing a 0.5 m × 0.5 m metacoating that maintains excellent optical uniformity and random structural characteristics (Fig. 4a). Cross-sectional SEM images taken at the top, middle and bottom regions of the ~ 100 μm thick metacoating show similar microstructures with randomly dispersed EZS in the K_2_SiO_3_ matrix, indicating a uniform structure without observable gradient features (Fig. 4b). EDS analysis confirms the compositional uniformity of the metacoating. The Zr and O signals delineate the EZS domains, while Eu shows a diffuse distribution due to its low doping concentration. Si is uniformly distributed, indicating the formation of a continuous three-dimensional silicate network, whereas slight local enrichment of K likely originates from K_2_SiO_3_ condensation (Fig. 4c). Infrared thermal imaging shows that the K_2_SiO_3_, EZS and metacoating appear markedly hotter than the surrounding bare Al, indicating high apparent emissivity arising from strong mid-infrared Si−O and Zr−O vibrations (Fig. 4d) [9]. Concurrently, the metacoating exhibits bright, uniform red luminescence from the Eu^3+ 5^D_0_ → ^7^F_2_ transition under 395 nm excitation (Fig. 4e).

To evaluate the radiative cooling performance of the EZS metacoating, we first compared its net cooling power with commercial and reported counterparts. As shown in Fig. 4f and Table S1, the EZS metacoating attains a maximum net cooling power of 323.69 W m^−2^, enabled by its ultralow *α*_*s*_ and *ε*, surpassing state-of-the-art counterparts that typically remain below 300 W m^−2^. In comparison, the widely used AZ-93 and YB-71 coatings, formulated with ZnO and Zn_2_TiO_4_ pigments, deliver net cooling powers of 212.89 and 253.79 W m^−2^, respectively. Their cooling performance is primarily limited by higher *α*_*s*_, which increases solar energy uptake and reduces overall cooling efficiency. Then the radiative cooling performance was tested in a vacuum chamber with a black-coated interior as the cold sink (Fig. 4g). To simulate the spacecraft thermal environment, each coated Al sheet was subjected to stable AM0 solar radiation (1367 W m^−2^) via a quartz window and fitted with a resistive heater, mimicking both solar exposure and internal heat generation (Fig. S19). Three independent measurements yielded standard deviations below 0.3 °C for the EZS metacoating and below 1 °C for the Al sheet, confirming the high stability of the testing system and the excellent reproducibility of the measurements (Fig. S20). Under AM0 solar illumination and 395 nm excitation, the EZS metacoating exhibits a bright irradiance spot and uniform red luminescence in Fig. 4h. For benchmarking, we compared it with TiO_2_ (*α*_*s*_ = 0.184, *ε* = 0.901), ZnO (*α*_*s*_ = 0.155, *ε* = 0.948) and Zn_2_TiO_4_ (*α*_*s*_ = 0.147, *ε* = 0.939) coatings, which represent the principal pigment systems used in above commercial all-inorganic radiative cooling coatings (Figs. S21 and S22) [29]. After 40 min of solar exposure, the EZS metacoating stabilizes at 28.1 °C, which is 16.6 °C cooler than bare Al sheet and cooler than TiO_2_, ZnO and Zn_2_TiO_4_ coatings by 7.3, 4.4 and 3.8 °C, respectively (Fig. 4i). With a 2 W internal heat load alone, the oxide-based coatings exhibit similar temperatures because their emittances are comparable, whereas the Al sheet heats rapidly due to its low *ε*. Under simultaneous solar exposure and internal heating, the EZS metacoating remains the top performer, operating 77.0 °C below bare Al and cooler than TiO_2_, ZnO and Zn_2_TiO_4_ coatings by 9.1, 5.2 and 3.8 °C. This superiority stems from its favorable photothermal balance (*α*_*s*_ = 0.076, *ε* = 0.931) and the optimized photonic architecture of the submicrosphere, which strengthens multiple backscattering to reject solar input while maintaining strong thermal emission to dissipate heat, thereby enabling highly efficient radiative cooling.

### Fluorescence Thermometry of EZS Metacoatings

**Fig. 5:**
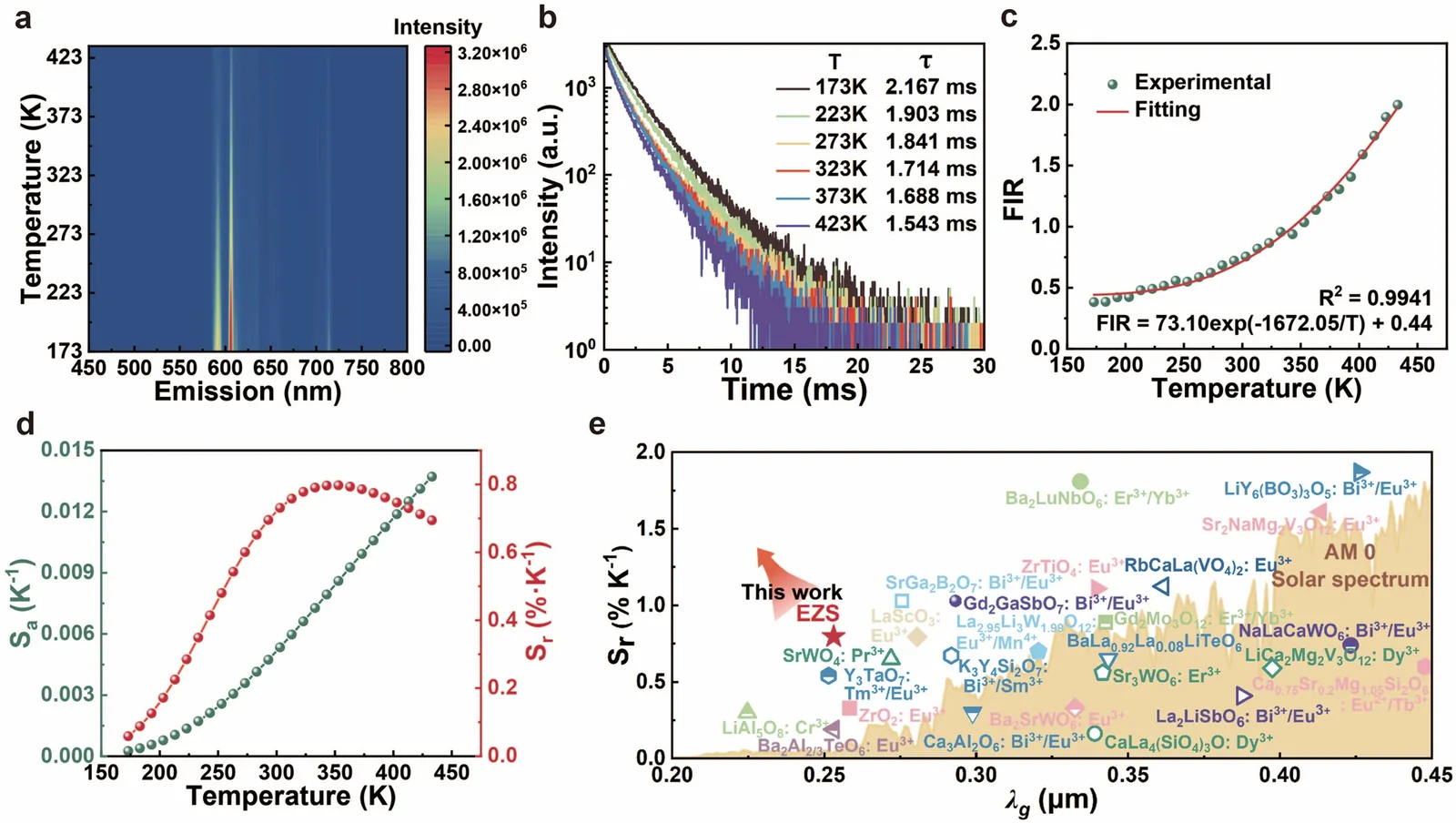
Temperature-dependent luminescence and thermometric performance of EZS metacoating. **a** Temperature-dependent emission spectra and **b** fluorescence decay curves of EZS under 395 nm excitation. **c** Variation of FIR (I_548_/I_606_) with temperature. **d** *S*_*a*_ and *S*_*r*_. **e** Comparison of *λ*_*g*_ and maximum *S*_*r*_ for the EZS metacoating and representative luminescent thermometric oxide

To assess the thermometric capability of EZS metacoatings, we measured temperature-dependent luminescence from 173 to 433 K. Under 395 nm excitation, the spectra display the characteristic Eu^3+^ emission bands (Fig. 5a). The integrated intensity decreases monotonically with increasing temperature, consistent with thermally activated nonradiative relaxation that lowers the radiative recombination efficiency (Fig. S23). In parallel, millisecond lifetimes indicate intrinsically low nonradiative decay, and their continuous shortening from 2.167 ms at 173 K to 1.543 ms at 423 K attests to the growing dominance of thermal quenching (Fig. 5b) [52]. The concurrence of a long baseline lifetime with pronounced thermal dependence is a hallmark of high-quality thermometric phosphors and underpins high signal-to-noise and sensitivity.

We further implemented FIR thermometry by using the intensity ratio I_548_/I_606_, corresponding to the ^5^D_1_ → ^7^F_2_ and ^5^D_0_ → ^7^F_2_ transitions, taking advantage of the stronger thermal quenching of the hypersensitive 606 nm line relative to the 548 nm emission, which generates a robust temperature-dependent fluorescence ratio. The FIR increases exponentially with temperature and is well described by a modified Boltzmann fit (*R*^2^ = 0.9941, Fig. 5c). The absolute sensitivity *S*_*a*_ rises to 0.0134 K^−1^ at 433 K, while the relative sensitivity *S*_*r*_ peaks at 0.797% K^−1^ at 353 K, indicating high resolution in the mid-temperature range and robust detectability at elevated temperatures (Fig. 5d). Benchmarking against reported thermometric oxides shows that the wide *E*_*g*_ of EZS shortens the *λ*_*g*_ and supports a low *α*_*s*_ of metacoating (Fig. 5e and Table S2). The EZS metacoating achieves higher *S*_*r*_ than materials with comparable or shorter *λ*_*g*_ (*S*_*r*_ typically below 0.55% K^−1^) and outperforms most high-*λ*_*g*_ systems, demonstrating its superior thermometric sensitivity and the effectiveness of a concurrently optimized material-structure strategy. Taken together, the results demonstrate broadband temperature responsiveness and high sensitivity over 173–433 K, highlighting the practical promise of low-*α*_*s*_ EZS metacoatings for integrated, non-contact optical thermometry and space radiative cooling.

### Irradiation Resistance in Extreme Space Environments

**Fig. 6:**
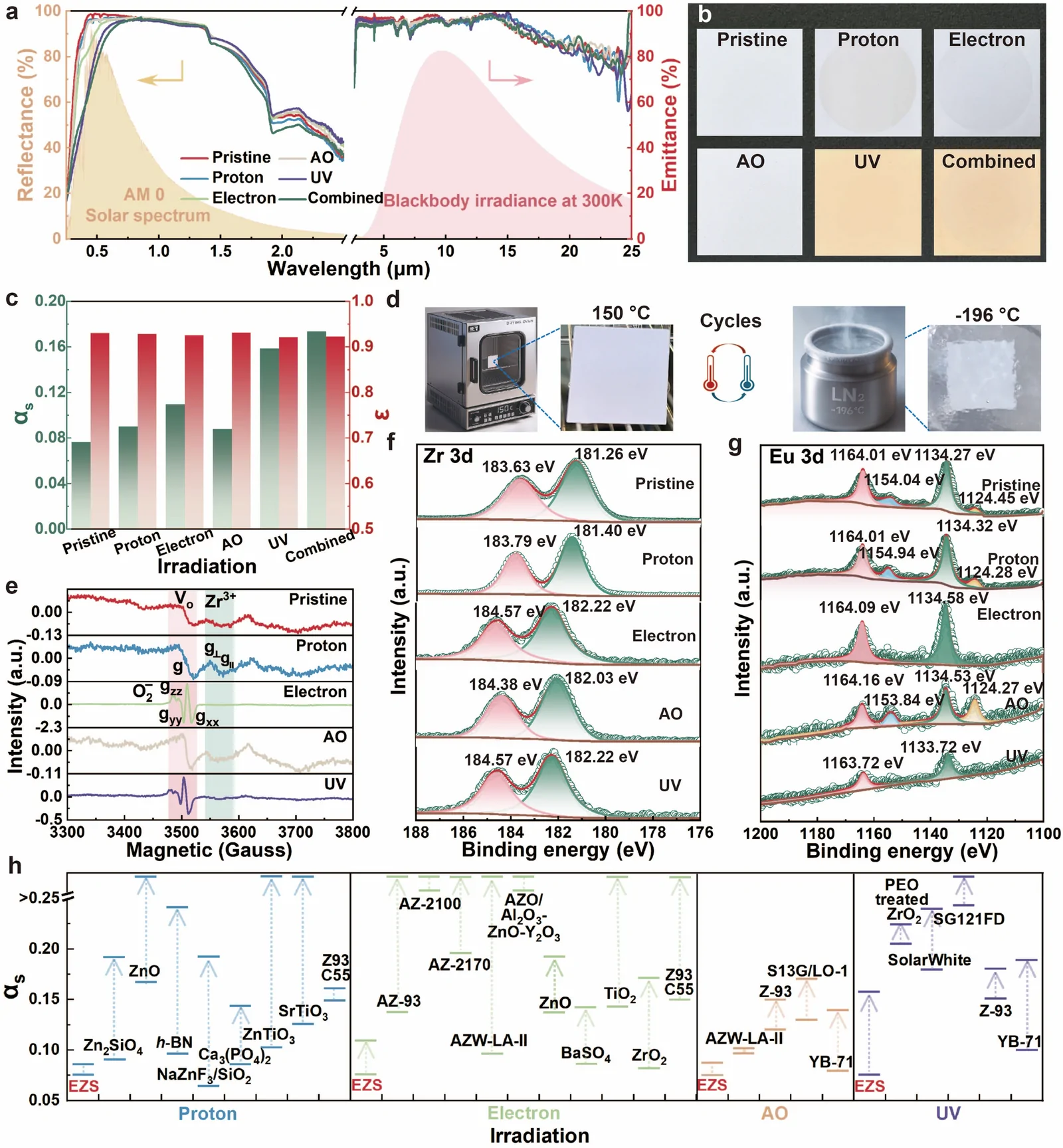
Irradiation resistance of the EZS metacoating under simulated space environments. **a** Reflectance and emittance spectra, **b** photographs of 4 cm × 4 cm samples and **c** comparison of *α*_*s*_ and *ε* before and after different irradiations. **d** Schematic and photographs after thermal cycles between -196 and 150 °C. **e** EPR spectra, **f** Zr 3*d* and **g** Eu 3*d* XPS spectra of the EZS metacoating before and after different irradiations. **h** Post-irradiation *α*_*s*_ comparison of EZS metacoating with other all-inorganic radiative cooling coatings after different irradiations

The suitability of a metacoating for long-term on-orbit service hinges on its resilience to space stressors, so post-irradiation optical stability is a primary metric. We measured the reflectance and emittance of the EZS metacoating after proton, electron, AO, UV and combined exposures (Figs. 6a, b). Proton and AO cause only slight losses in 0.25–0.70 µm with minimal color change. Electron caused a pronounced reflectance to drop in the 0.38–0.70 µm range and produced a gray tint, indicating the formation of color centers that absorb visible light as a result of irradiation-induced lattice damage. UV irradiation produces a marked reflectance drop across 0.25–1.38 µm together with a light ochre tint, attributable to photoinduced charging and the creation of surface states that increase absorption. The metacoating exhibits surface coarsening and locally fragmented features after UV and combined irradiations (Fig. S24), likely arising from photo-induced bond scission within the silicate network. Nevertheless, the matrix remains continuous and effectively anchors the EZS. Across all cases, changes in the infrared are minor. Consistently, the *α*_*s*_ shifts from 0.076 to 0.090 (proton), 0.110 (electron), 0.088 (AO), 0.159 (UV) and 0.174 (combined irradiations), while the *ε* is essentially unchanged with variations below 0.01 (Fig. 6c). Although the *α*_*s*_ increases noticeably after UV and combined irradiations, the metacoating still delivers net cooling powers of 206.55 and 185.59 W m^−2^ at 300 K, respectively, demonstrating that effective radiative cooling performance is retained. Thermal cycling is equally critical for space reliability. After 50, 100, and 150 thermal cycles between -196 and 150 °C, the *α*_*s*_ increases by 0.009, 0.016, and 0.032, and *ε* decreases by 0.002, 0.007, and 0.015, accompanied by gradually enlarged microcracks, while the optical properties remain largely stable, demonstrating good thermal shock tolerance (Figs. 6d, S25, and S26).

A comparative evaluation with representative radiative cooling coatings demonstrates that the EZS metacoating maintains the lowest *α*_*s*_ in the cohort before and after electron, proton, AO, UV and combined exposures (Fig. 6h and Table S3). In particular, compared with the widely used AZ-93 and YB-71 coatings, the EZS metacoating preserves an *α*_*s*_ advantage exceeding 0.04 even after proton, AO and UV irradiations. Meanwhile, the fluorescence thermometric performance remains well preserved after combined irradiations, with a maximum temperature deviation below 6.1%, owing to the intrinsic robustness of FIR thermometry and the high spectral stability of Eu^3+^ emissions (Fig. S27). Pull-off tests reveal strong adhesion between the EZS metacoating and the Al sheet, with strengths of 2.2 and 1.5 MPa before and after combined irradiations, respectively (Fig. S28). SEM images further confirm a stable interface between metacoating and Al sheet without noticeable delamination after combined irradiations, demonstrating stable adhesion and structural integrity (Fig. S29). These findings confirm that the EZS metacoating possesses exceptional irradiation resistance, sustaining its low *α*_*s*_, high *ε* and stable fluorescence thermometry under multiple space stressors, thereby underscoring its strong potential for long-term and stable spacecraft thermal management.

Optical changes following irradiations primarily originate from defect formation. We employed electron paramagnetic resonance (EPR) spectroscopy to analyze the EZS metacoating before and after exposure to proton, electron, AO and UV irradiations (Fig. 6e). The pristine metacoating exhibits two anisotropic signals at ${g}_{\perp }$ = 1.9751 and ${g}_{\parallel }$ = 1.9598, consistent with a low concentration of Zr^3+^ centers, along with an isotropic signal at g = 2.0027 characteristic of singly charged* V*_*O*_. After proton and AO irradiations, the intensities of both the Zr^3+^ and *V*_*O*_ signals increase slightly, suggesting the formation of defects at relatively low concentrations. This limited defect generation is consistent with the small increase in *α*_*s*_ observed after these irradiations. In contrast, electron and UV irradiations generate a rhombic triplet at ${g}_{zz}$= 2.0142, ${g}_{yy}$ = 2.0048, ${g}_{xx}$ = 1.9947 and ${g}_{zz}$ = 2.0152, ${g}_{yy}$ = 2.0059, ${g}_{xx}$ = 1.9956, characteristic of O^−^ centers [53, 54]. These g-tensors arise when holes created by excitation of valence-band electrons are trapped by lattice O^2−^ under irradiation [55]. The formation of these defect centers introduces localized *E*_*g*_ states that act as color centers and enhance solar absorption, while the stronger EPR signal intensity observed after UV irradiation compared with electron irradiation indicates a higher defect concentration, consistent with the more pronounced increase in *α*_*s*_.

XPS characterization further corroborates the EPR findings. In the Zr 3*d* region, the pristine metacoating shows Zr^4+^ peaks at 181.26 eV (3*d*_5/2_) and 183.63 eV (3*d*_3/2_) with a spin–orbit splitting of ~ 2.37 eV (Fig. 6f). Proton and AO exposures induce minor binding energy shifts. In contrast, electron and UV irradiations shift the Zr 3*d* doublet to 182.22 and 184.57 eV while maintaining Zr^4+^ character (splitting ~ 2.35 eV), suggesting a net reduction of electron density due to nearby defect-charge rearrangement. The Eu 3*d* spectra display characteristic Eu^3+^ peaks at 1134.27 and 1164.01 eV (Δ*E* = 29.59 eV) in Fig. 6g, accompanied by satellites at 1124.45 and 1154.04 eV reflecting enhanced 4*f* localization [44, 45]. While proton and AO irradiations cause only negligible spectral changes, electron and UV exposures shift the Eu 3*d* peaks to 1134.58/1164.09 and 1133.72/1163.72 eV, respectively, accompanied by a near disappearance of the satellite features. The suppression of these satellites indicates partial delocalization of Eu^3+^ 4*f* electrons and the participation of Eu^3+^ 4*f* states in irradiation-induced charge-transfer processes. Despite these electronic perturbations, the modest energy shifts confirm that Eu remains predominantly in the trivalent state, suggesting that irradiation alters the local electronic environment rather than the oxidation state. Similar perturbations are observed in O, K and Si spectra, consistent with local bonding rearrangements (Fig. S30). Meanwhile, FTIR and XRD show no discernible changes, confirming the phase and chemical composition remain essentially intact (Fig. S31).

## Conclusions

In summary, we have demonstrated a dual-functional photonic EZS metacoating that integrates high-performance space radiative cooling with high-sensitivity fluorescence thermometry. Guided by photonic-scattering optimization and supported by controlled synthesis of tetragonal EZS with independently tuned diameter and Eu content, the optimal configuration with 8.48% Eu doping, 0.756 µm diameter and 35% volume fraction, achieves strong Eu^3+^-centered emission and minimal *α*_*s*_. Combined DFT and experimental analyses reveal that localized Eu^3+^ 4*f* states govern efficient luminescence without largely narrowing the *E*_*g*_, maintaining low *α*_*s*_. The resulting metacoating exhibits *α*_*s*_ = 0.076 and *ε* = 0.931, yielding a net cooling power of 323.69 W m^−2^. Under simulated vacuum space conditions, it reduces the temperature of an Al sheet by approximately 77 °C and operates 9.1, 5.2 and 3.8 °C cooler than TiO_2_, ZnO and Zn_2_TiO_4_ coatings, respectively. Its fluorescence enables contactless temperature sensing over a wide range of 173–433 K, exhibiting a maximum absolute sensitivity of *S*_*a*_ = 0.0134 K^−1^ and a relative sensitivity of *S*_*r*_ = 0.797% K^−1^. The wide *E*_*g*_ of EZS, together with the large Stokes shift of Eu^3+^ emission, ensures reliable thermometric operation under AM0 illumination without compromising radiative cooling performance. The metacoating maintains the lowest *α*_*s*_ and a stable *ε* after proton, electron, AO, UV and combined irradiations, outperforming commercial and literature-reported coatings and underscoring its irradiation resistance, while EPR/XPS analyses attribute the slight coloration to *V*_*O*_ and O⁻ defects. Collectively, the EZS metacoating, combining radiative heat rejection, self-perceptive temperature readout and irradiation robustness, provides a scalable and durable platform for intelligent spacecraft thermal management, offering a blueprint for coatings capable of autonomously monitoring and regulating thermal loads in orbit.

## Supplementary Information

Below is the link to the electronic supplementary material.

Supplementary file1 (DOCX 12672 KB)
