# Supplementary material for: Dual-Functional Photonic Metacoating Integrating Fluorescence Thermometry and High-Performance Space Radiative Cooling
Source: Nanomicro Lett. 2026 Apr 29;18:349. doi: 10.1007/s40820-026-02195-8 (PMC13129188; doi:10.1007/s40820-026-02195-8)
Supplement: Supplementary file 1 — Supplementary file1 (DOCX 12672 KB) [file 40820_2026_2195_MOESM1_ESM.docx]

Supporting Information for

**Dual-Functional Photonic Metacoating Integrating Fluorescence Thermometry and High-Performance Space Radiative Cooling**

Hao Gong^1^, Zhongyang Wang^1,^ *, Yan Zheng^1^, Liping Tong^2^, Hongchao Li^1^, Zhiyuan Zhao^1^, Junjia Liu^1^, Gang Liu^3^, Xiao Zhou^1,^ *, Tongxiang Fan^1,^ *

^1^State Key Laboratory of Metal Matrix Composites, School of Materials Science and Engineering, Shanghai Jiao Tong University, Shanghai 200240, P. R. China

^2^School of Materials, Shanghai Dianji University, Shanghai, 201306, P. R. China

^3^Shanghai Institute of Spacecraft Equipment, Shanghai, 200240, P. R. China

*Corresponding authors. E-mail: zy_wang@sjtu.edu.cn (Zhongyang Wang); zhouxiao113@sjtu.edu.cn (Xiao Zhou); txfan@sjtu.edu.cn (Tongxiang Fan)

# **S1 Supplementary Experiment Section**

**S1.1 Chemicals**

Zirconium n-propoxide (Zr(OC_3_H_7_)_4_, 70% in n-propanol, stored in a glovebox), ethanol (C_2_H_5_OH, ≥99.8%, chromatographic grade), stearic acid (C_17_H_35_COOH, 98%), triethylamine (C_6_H_15_N, 99%), potassium silicate (K_2_O·nSiO_2_·xH_2_O), europium nitrate hexahydrate (Eu(NO_3_)_3_·6H_2_O, 99.99%) were purchased from Shanghai Aladdin Biochemical Technology Co., LTD.

**S1.2 Morphology, Structural and Compositional Characterizations**

Surface morphologies were investigated by scanning electron microscopy (SEM, TESCAN RISE-MAGNA, Czech Republic) operated at 10 kV. Elemental distributions were examined by energy-dispersive X-ray spectroscopy (EDS, Oxford X-Max N20, UK) attached to the SEM. Submicrosphere size distributions and standard deviations were obtained using Nano Measurer software.

Microstructural and compositional analyses were carried out by high-resolution transmission electron microscopy (HR-TEM, Talos F200X, Thermo Fisher Scientific, USA) at 200 kV. Elemental mapping was conducted with a Super-X EDS detector in high-angle annular dark-field scanning TEM (HAADF-STEM) mode. Internal architectures were examined on ultrathin sections prepared at room temperature using an ultramicrotome (Leica EM UC7FC7, Germany). Atomic-resolution images were acquired on an aberration-corrected STEM (AC-STEM, JEM-ARM200F, JEOL, Japan) with a cold field-emission gun operated at 200 kV. EDS was performed with a JEOL JED-2300 detector using a probe convergence angle of 20.6 mrad and a collection semi-angle of 54-220 mrad.

The crystal structure was characterized by X-ray diffraction (XRD, Rigaku D/Max 2550 PC, Japan) using Cu Kα radiation (λ = 1.5406 Å) over 2θ = 10-80°, with an operating voltage of 45 kV and current of 200 mA.

FTIR spectra were recorded either in transmission mode or using an attenuated total reflection (ATR) accessory (Thermo Fisher Nicolet S6700, USA) over 4000-400 cm^-1^, each spectrum was averaged over 64 scans.

Eu and Zr contents were quantified by inductively coupled plasma optical emission spectroscopy (ICP-OES, iCAP PRO, Thermo Fisher Scientific, USA) after dissolution in hydrofluoric acid.

X-ray photoelectron spectroscopy (XPS, Thermo Scientific K-Alpha, USA) was used to probe the chemical states of O, Zr and Eu with a monochromatic Al Kα source (*hν* = 1486.8 eV, 12 kV, 6 mA). Spectra were collected at a pass energy of 50 eV with 0.1 eV step size under a base pressure of ~5 × 10^-7^ Pa, and all binding energies were referenced to the C 1s peak at 284.8 eV.

Electron paramagnetic resonance (EPR) measurements were performed on a Bruker EMXplus-9.5/12 spectrometer (Germany) operating at X-band (9.826 GHz) with 100 kHz field modulation. Data were acquired at room temperature with a modulation amplitude of 0.5 G and a microwave power of 200 mW.

Infrared radiative behavior was assessed using a thermal imaging camera (Guide Sensmart PT650, China) with a 640 × 480 pixel uncooled vanadium oxide detector, operating at 30 Hz. The instrument covers a temperature range of -40 to 150 °C with a thermal sensitivity of 30 mK, an accuracy of ±1 °C, and a spectral response of 7.5-14 µm. The emittance was set to 0.92. Thermal images and videos were processed using Guide ThermoTools software.

**S1.3 Fluorescence Lifetime and Thermometric Sensitivity Calculation**

Fluorescence intensity is proportional to the population of ions in the excited state. After the excitation source is switched off, the emission decays with time, and the average fluorescence lifetime (*τ*) is defined as the time at which the intensity has decreased to 1/e of its initial value. The decay curves *I*(*t*) were fitted with a biexponential function [S1, S2]:

$I(t)=I_{0}+I_{1}e^{-t/\tau_{1}}+I_{2}e^{-t/\tau_{2}}$ (S1)

where $\tau_{1}$ and $\tau_{2}$represent the fast and slow decay components, *I*_1_ and *I*_2_ denote their amplitudes, and *I*_0_ is the residual background. The mean lifetime was calculated as:

$\tau=\frac{\sum I_{n}\tau_{n}^{2}}{\sum I_{n}\tau_{n}}$ (S2)

The absolute sensitivity (*S_a_*) of the fluorescence intensity ratio (FIR) thermometry was evaluated from the Boltzmann-type relationship [S3, S4]:

$S_{a}=\frac{I_{548}}{I_{606}}=A\exp(-\frac{\Delta E}{k_{B}T})$ (S3)

where *I*_548_ and *I*_606_ are the integrated intensities corresponding to the ^5^D_1_→^7^F_2_ and ^5^D_0_→^7^F_2_ transitions of Eu^3+^, respectively. *A* is a proportionality constant related to the spontaneous emission probability and photon frequency, Δ*E* denotes the energy gap between the two thermally coupled levels, and *k*_B_ is the Boltzmann constant.

Relative sensitivity (*S_r_*) is a key figure of merit for luminescent thermometers, is defined as:

$S_{r}=\left| \frac{1}{FIR}\frac{ⅆFIR}{ⅆT} \right|\times100\%=\frac{\Delta E}{k_{B}T}\times100\%$ (S4)

These analyses enable a quantitative assessment of the luminescent and thermometric performance of EZS and the corresponding metacoatings.

**S1.4 Data analysis**

The solar absorptance (*α*_s_) was evaluated from its measured reflectance *R*(*λ*) under the AM0 solar spectrum (*I*_AM0_(*λ*)) as:

$\alpha_{s}=\frac{\int_{0.25\text{μm}}^{2.5\text{μm}} (1-R\left(\lambda\right))I_{AM0}\left(\lambda\right)d\lambda}{\int_{0.25\text{μm}}^{2.5\text{μm}} I_{AM0}\left(\lambda\right)d\lambda}$ (S5)

The hemispherical emittance (*ε*) was obtained using Kirchhoff’s law, assuming that in the mid-infrared the emittance equals the spectral absorbance (*α*(*T*, *λ*)):

(S6)

where the ideal blackbody spectral radiance (*I*_b_(*λ, T*)) is:

$I_{b}\left(T,\lambda\right)=\frac{2hc^{2}}{\lambda^{5}}\frac{1}{\exp(\frac{hc}{k\lambda T})-1}$ (S7)

Here, *h* is Planck’s constant, *k* is Boltzmann’s constant, and *c* is the speed of light in vacuum.

**S1.5 Calculations of net cooling power**

The net radiative cooling power was calculated as:

𝑃_𝑛𝑒_*_t_*(𝑇) = 𝑃_𝑟𝑎𝑑_(𝑇) − 𝑃_𝑠𝑜𝑙𝑎𝑟_ (S8)

with the radiative power exchanged with deep space (background temperature, *T_bg_* ≈ 3K) given by:

$P_{\text{rad}}(T)=\varepsilon\sigma(T^{4}-T_{\text{bg}}^{4})$ (S9)

where (*σ* = 5.67 × 10^-8^ W·m^-2^·K^-4^) is the Stefan-Boltzmann constant and *T* is the surface temperature. The absorbed solar power was evaluated as:

$P_{\text{solar}}=\alpha_{s}S\cos(\theta)$ (S10)

where *α_s_* is the solar absorptance, *S* = 1367 W·m^-2^ is the solar constant, and *θ* is the solar incidence angle. These expressions were used to compare the net cooling performance of the metacoating with other all-inorganic radiative cooling coatings and to assess their suitability for spacecraft thermal management.

**S2 Supplementary Figures and Tables**


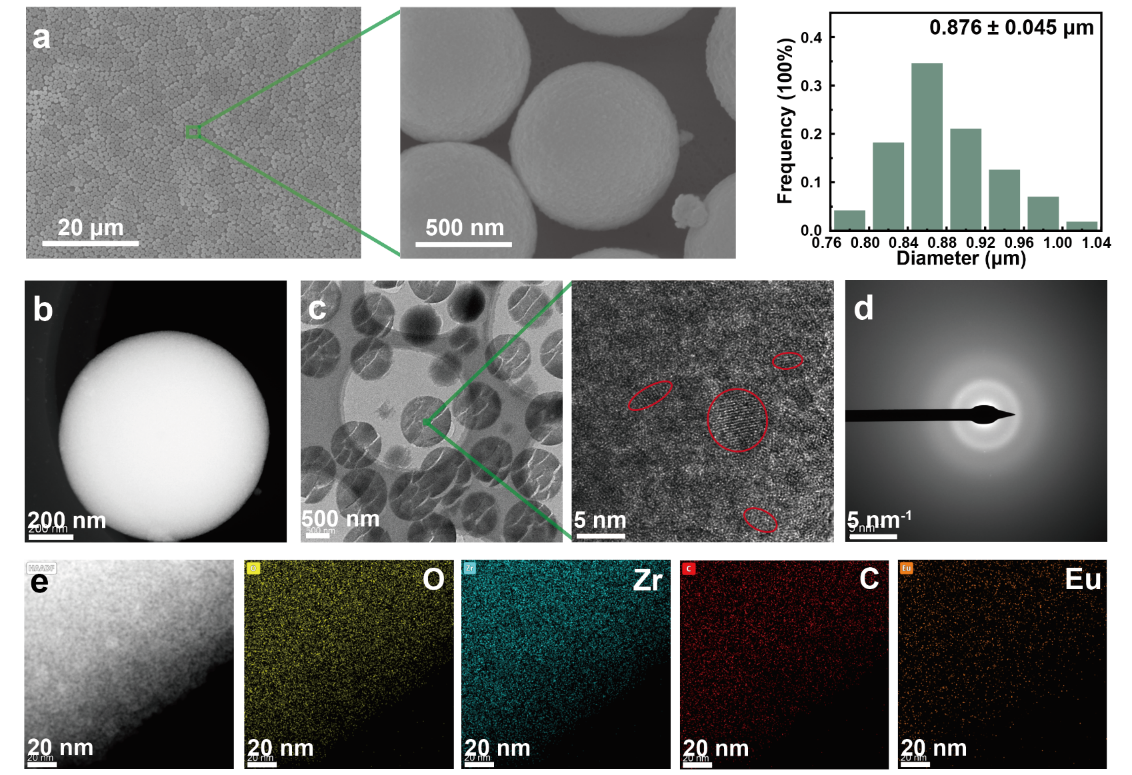


**Fig. S1** Morphology and composition of EZS precursors synthesized by forced alkoxide hydrolysis with concurrent Eu^3+^ coordination under hydrothermal conditions. **a** Low- and high-magnification SEM images showing uniform spherical particles with an average diameter of 0.876 μm. **b** HAADF-TEM image confirming a symmetric spherical morphology. **c** HR-STEM image revealing a predominantly disordered interior with only localized nanocrystalline fringes a few nanometers in size. **d** SAED pattern without discernible diffraction rings, consistent with an amorphous precursor. **e** HAADF-STEM and EDS elemental maps (O, Zr, C, Eu) demonstrating homogeneous elemental distributions and absence of Eu segregation, indicative of uniform Eu^3+^ incorporation within the ZrO_2_ submicrosphere precursor


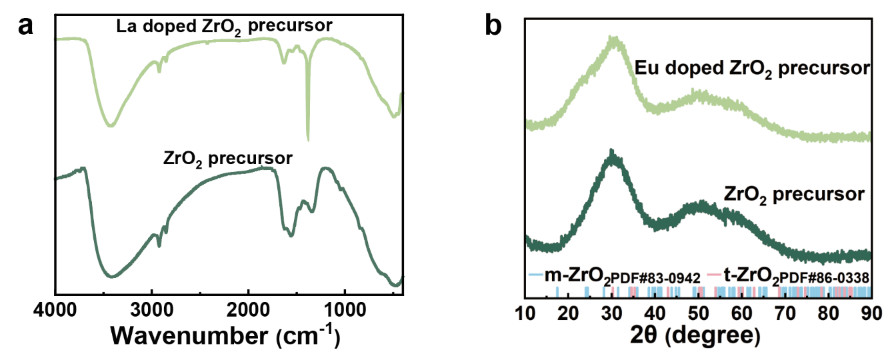


**Fig. S2 a** FTIR spectra and **b** XRD patterns of EZS and ZrO_2_ submicrosphere precursors

FTIR and XRD analyses were performed to examine the chemical composition and structural evolution of EZS precursors. Both EZS and ZrO_2_ submicrosphere precursors exhibit characteristic absorption peaks of organic functional groups, indicating residual organics in the as-prepared samples. After hydrothermal diffusion doping, the Zr−OH stretching vibrations at 3400-3560 cm^-1^ weaken significantly, suggesting coordination between Eu^3+^ ions and surface Zr−OH groups [S5]. The alkyl stretching bands at 2920 cm^-1^ and 2850 cm^-1^, as well as the bending mode at 1460 cm^-1^, are notably reduced, confirming that hydrothermal treatment promotes the hydrolysis and removal of propoxy groups [S6]. The sharper absorption bands between 400-600 cm^-1^ correspond to metal-oxygen bond stretching, while the new peak at 1386 cm^-1^ arises from the NO_3_^−^ groups of europium nitrate solution. The XRD patterns of both EZS and ZS precursors display broad diffraction features, consistent with an amorphous structure. Together with HR-TEM observations, these results confirm that the EZS precursors remain predominantly amorphous after hydrothermal processing.


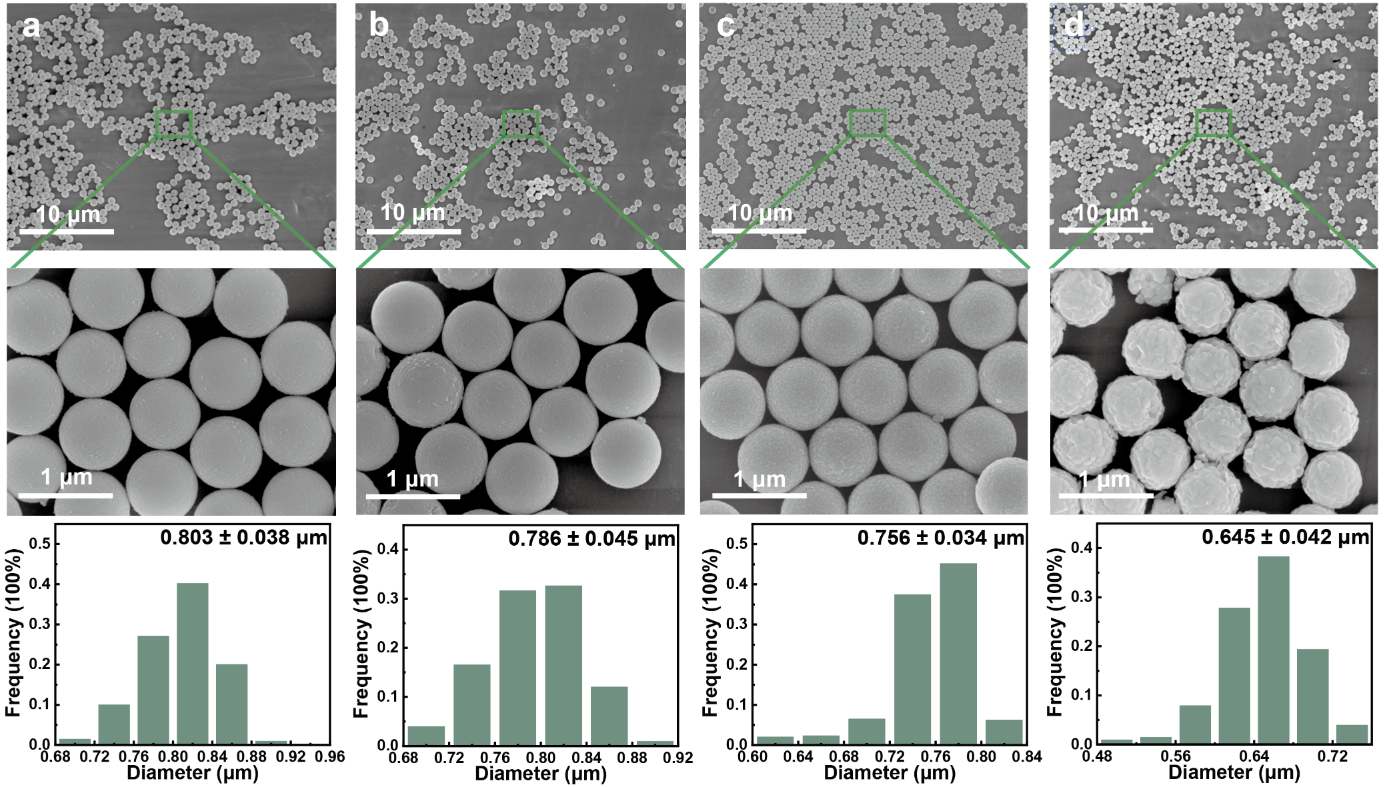


**Fig. S3** Low- and high-magnification SEM images and corresponding diameter distributions of EZS precursors after calcination at different temperatures: **a** 600 °C, **b** 800 °C, **c** 1000 °C and **d** 1200 °C

To obtain highly crystalline and spherical EZS, we examined the effects of calcination on diameter and microstructure. As shown in Fig. S3, calcination effectively removes organics from the precursors and promotes crystallization. At 600 °C, the average diameter decreases to 0.803 µm. Further calcination at 800 °C and 1000 °C reduces the diameter to 0.786 µm and 0.745 µm, accompanied by increased surface roughness. At 1200 °C, enhanced atomic diffusion produces faceted grains and a denser surface, with the diameter decreasing to 0.645 µm, indicative of grain growth and particle densification at high temperature. Across the studied range, the EZS retain a spherical morphology up to 1200 °C, while exhibiting a monotonic size reduction with increasing temperature.


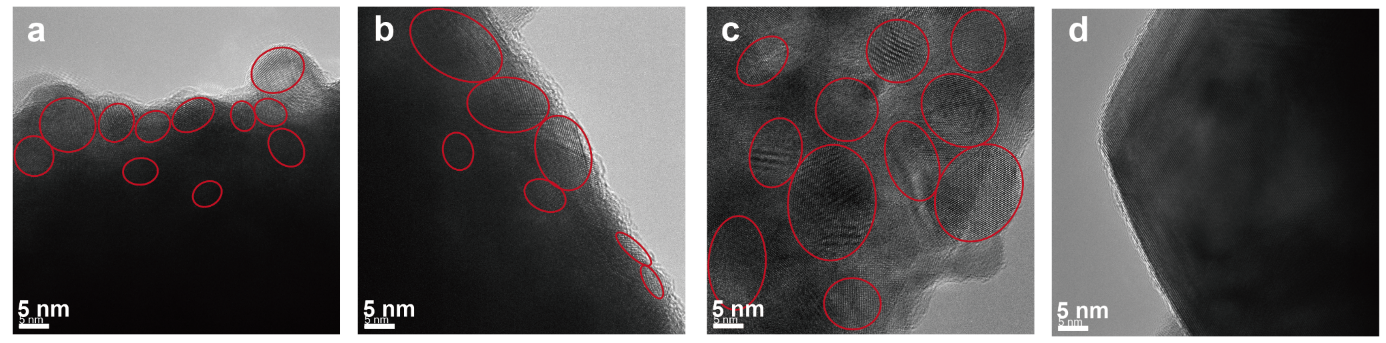


**Fig. S4** HR-TEM images of EZS after calcination at different temperatures: **a** 600 °C, **b** 800 °C, **c** 1000 °C and **d** 1200 °C

HR-TEM analysis was performed to study the evolution of grain structure at the edge of EZS after heat treatment at different temperatures, as shown in Fig. S4. After calcination at 600 °C, a large number of small primary grains appeared at the edges of the EZS. As the temperature increased to 1000 °C, the crystal growth accelerated under thermal driving, resulting in noticeably larger grains. At 1200 °C, the grain size further increased to several tens of nanometers, with significantly improved crystallinity and structural integrity. The edge regions exhibited a trend toward densification, which is closely related to the enhanced atomic diffusion and grain fusion induced by high temperatures.


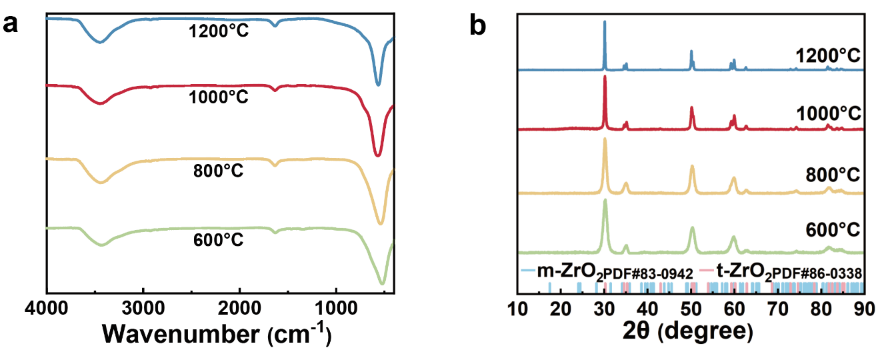


**Fig. S5** **a** FTIR spectra and **b** XRD pattern of EZS after calcination at different temperatures

FTIR spectroscopy and XRD partterns were employed to characterize the chemical composition and crystal structure of the EZS after calcination. As shown in Fig. S5a, the FTIR spectra reveal a noticeable weakening and smoothing of the absorption peaks in the 900-4000 cm^-1^ range at 600 °C, indicating the removal of most organic components. When the heat treatment temperature was increased from 600 °C to 1200 °C, the FTIR peaks in the 750-400 cm^-1^ region became sharper, suggesting an improvement in crystallinity. These peaks correspond to localized bending and deformation vibration modes of metal-oxygen bonds. As shown in Fig. S5b, the XRD results indicate that the EZS transition from an amorphous state to a tetragonal phase after calcination. At 600 °C, characteristic diffraction peaks appear at 2θ = 30.1°, 35.1°, 50.3° and 59.9°, corresponding to the (101), (110), (112) and (211) planes of tetragonal ZrO_2_ (PDF#86-0338) [S7]. When the temperature is increased to 1000 °C and 1200 °C, these diffraction peaks become sharper, indicating a significant improvement in crystallinity. Additionally, secondary diffraction peaks appear at 2θ = 34.5°, 50.5° and 59.2°, corresponding to the (002), (200) and (103) planes of tetragonal ZrO_2_, respectively.


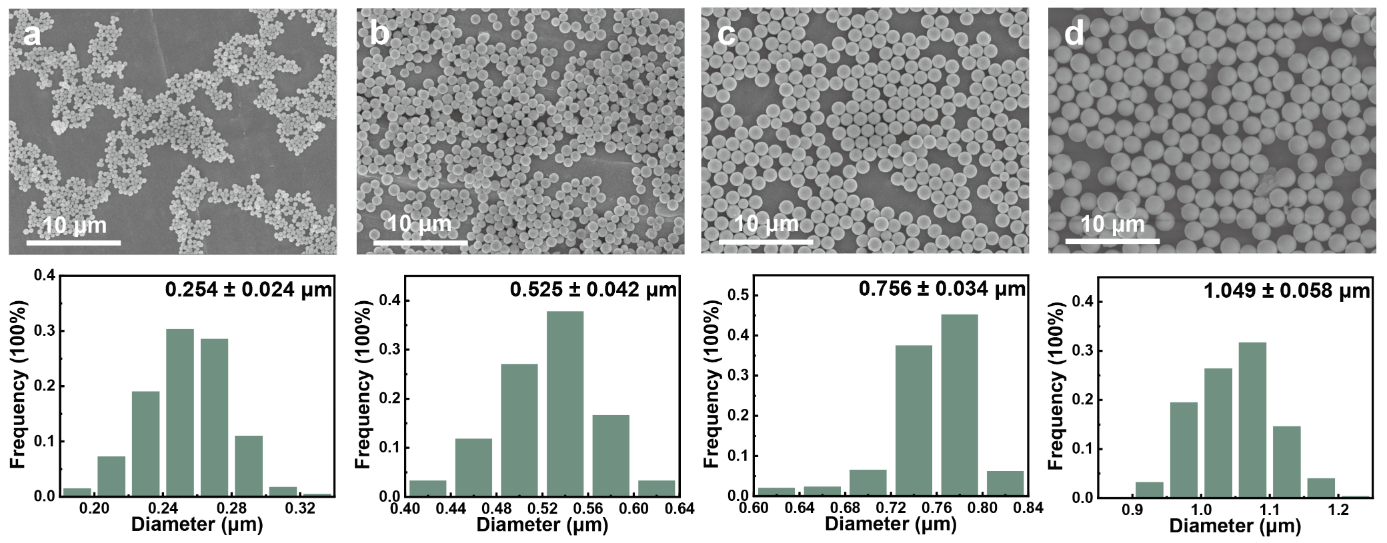


**Fig. S6** SEM images of EZS with different diameters: **a** 0.254 μm, **b** 0.525 μm, **c** 0.756 μm and **d** 1.049 μm


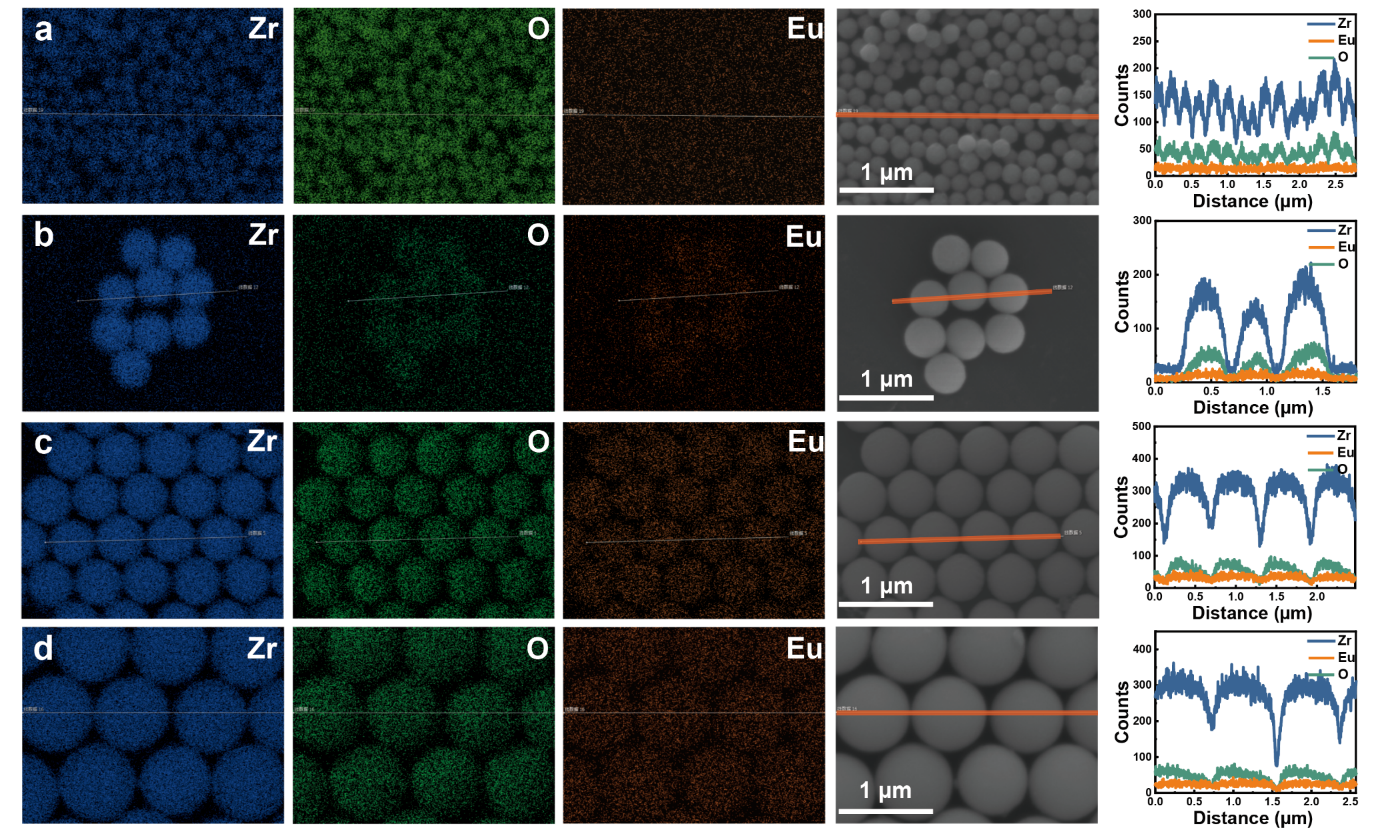


**Fig. S7** The SEM images, Zr, O and Eu elemental EDS-mapping and line scanning of EZS with different diameters: **a** 0.254 μm, **b** 0.525 μm, **c** 0.756 μm and **d** 1.049 μm.


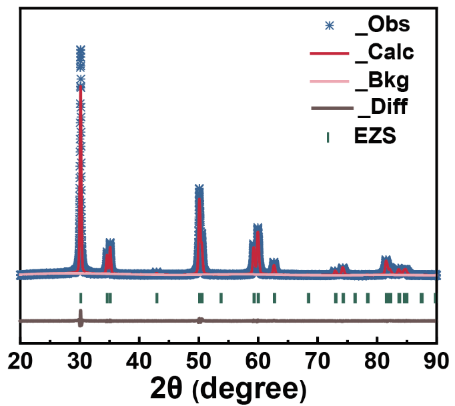


**Fig. S8** Rietveld refinement of the XRD pattern for EZS


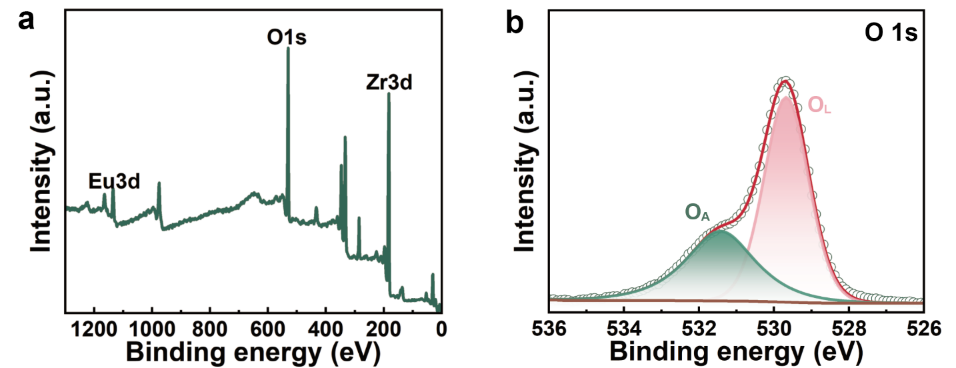


**Fig. S9** XPS **a** survey and **b** O 1s high-resolution spectra of EZS


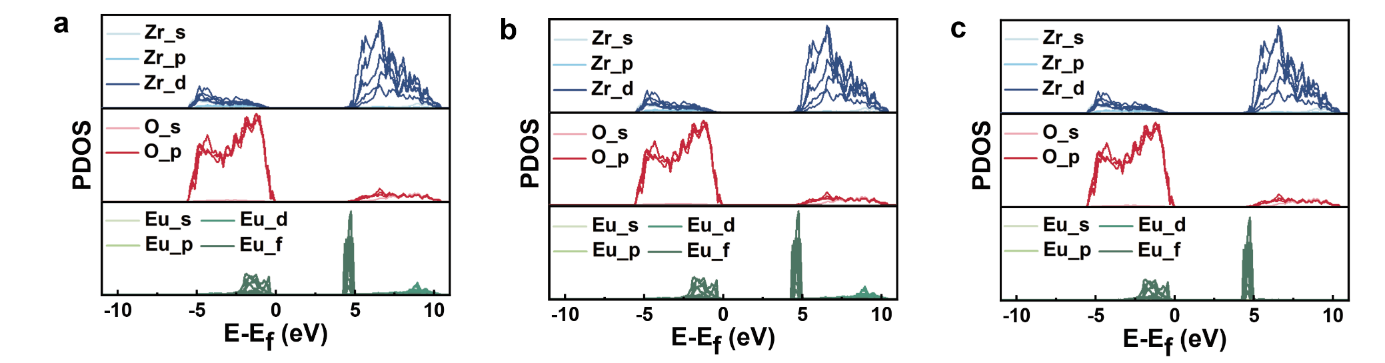


**Fig. S10** PDOS of **a** 2.78, **b** 4.17% and **c** 12.50% Eu-doped ZrO_2_


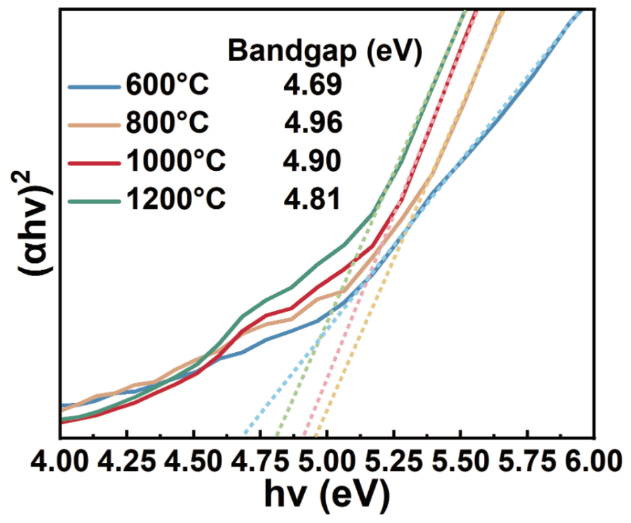


**Fig. S11** Optical *E_g_* of EZS after calcination at different temperatures

The optical *E_g_* of EZS was determined from UV-Vis absorption spectra using Tauc-plot analysis. As the calcination temperature increased from 600 to 800 °C, *E_g_* broadened from 4.69 to 4.96 eV, indicating improved crystallinity and a reduced defect density. When the temperature reached 1200 °C, *E_g_* decreased to 4.81 eV, which is attributed to the higher concentration of *V_O_* defects associated with aliovalent doping. These impurity states introduce defect levels, thereby narrowing the apparent *E_g_*.

**
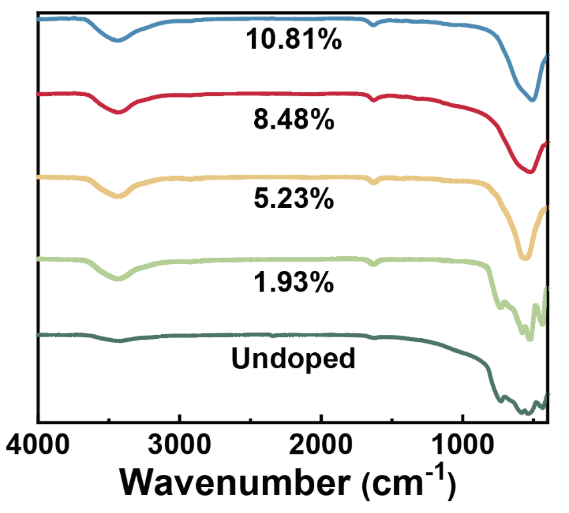
**

**Fig. S12** FTIR spectra of EZS at different doping concentration


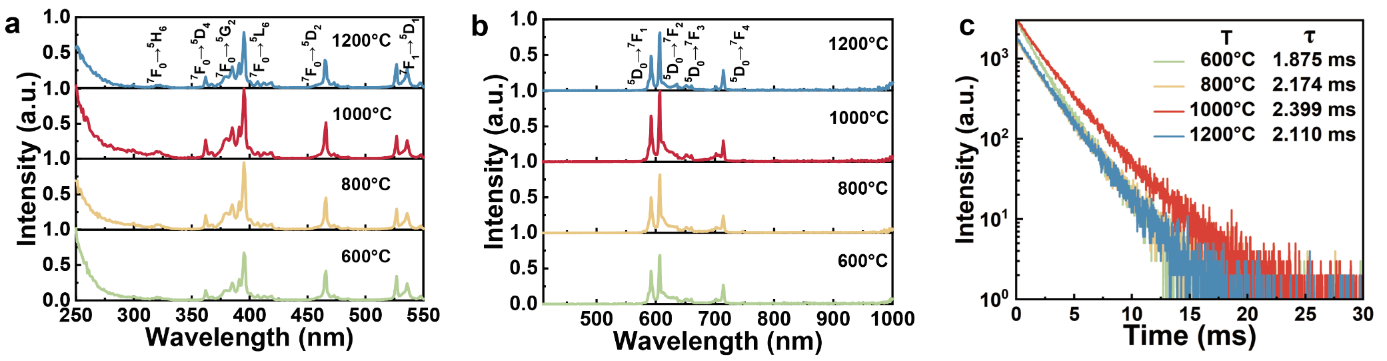


**Fig. S13** **a** Excitation and **b** emission spectra, and **c** fluorescence decay curves of EZS after calcination at different temperatures recorded at λ_em_ = 606 nm and λ_ex_ = 395 nm

With increasing calcination temperature, the crystallinity of the EZS gradually improves, grain size enlarges, and the local coordination environment of Eu^3+^ becomes more stable, leading to a continuous enhancement in emission intensity. The sample treated at 1000 °C exhibits the strongest luminescence, while a slight decrease occurs at 1200 °C due to the generation of additional *V_O_*, which act as quenching centers that trap excitation energy and reduce emission efficiency. Fig. S13c presents fluorescence decay curves recorded at an excitation wavelength of 395 nm and an emission wavelength of 606 nm. All samples exhibit lifetimes on the millisecond scale, indicating partially forbidden transitions. The decay profiles fit a biexponential model with time constants of 1.875, 2.174, 2.399 and 2.110 ms, respectively. The lifetime first increases and then decreases with temperature, which is attributed to changes in local symmetry. Moderate calcination temperature enhances structural order and suppresses nonradiative decay, while higher temperatures introduce lattice defects that open additional nonradiative channels, thus shortening the lifetime. Considering both excitation and emission behavior, 1000 °C is identified as the optimal annealing condition for achieving the highest luminescence performance.

**
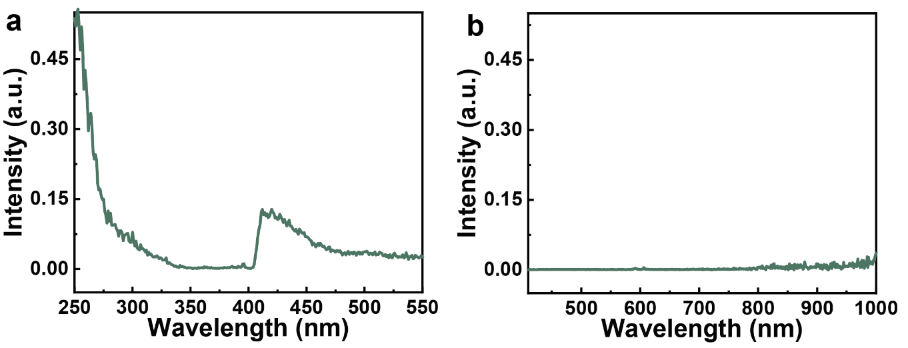
**

**Fig. S14 a** Excitation and **b** emission spectra of undoped ZrO_2_ submicrosphere recorded at λ_em_ = 606 nm and λ_ex_ = 395 nm

**
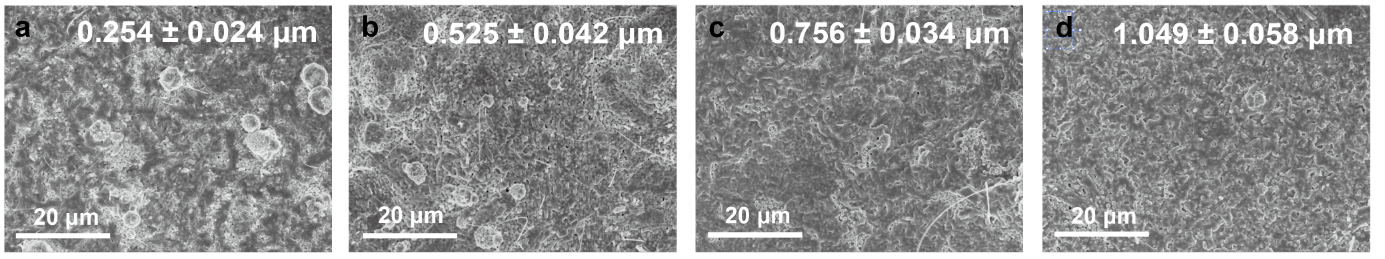
**

**Fig. S15** The low-magnification SEM images of metaoating with different EZS diameter: **a** 0.254 μm, **b** 0.525 μm, **c** 0.756 μm and **d** 1.049 μm

**
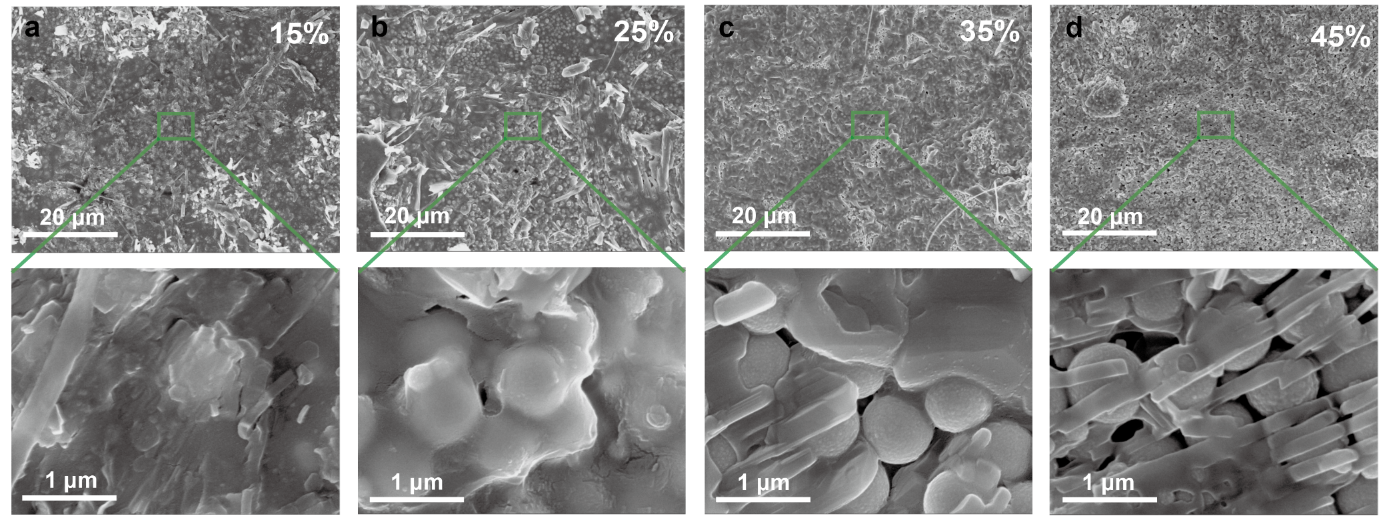
**

**Fig. S16** The low and high-magnification SEM images of metaoating with different volume fraction: **a** 15%, **b** 25%, **c** 35% and **d** 45%

**
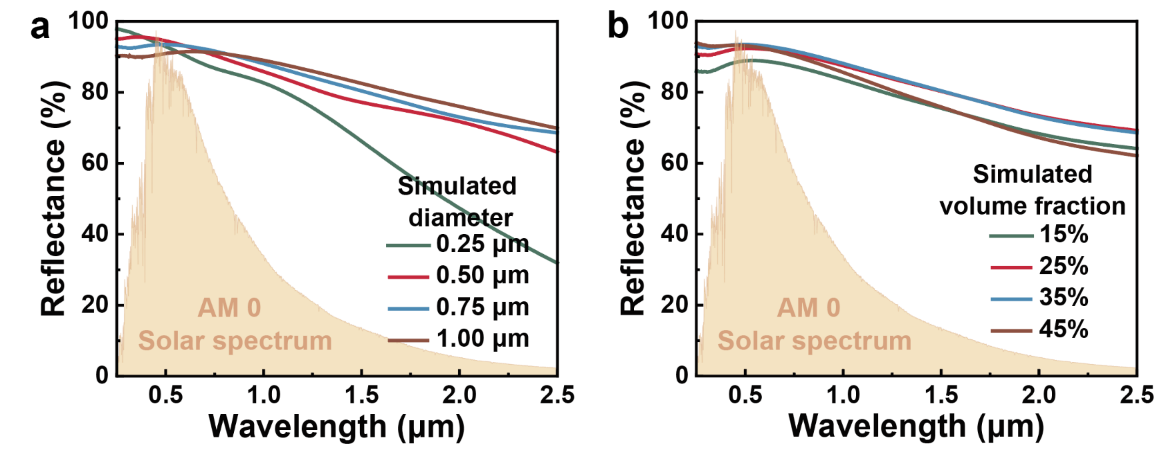
**

**Fig. S17** Simulated reflectance spectra of metacoating with different **a** EZS diameter and **b** volume fraction. The typical experimental parameters are a diameter of 0.75 μm, a volume fraction of 35% and a thickness of 100 μm

**
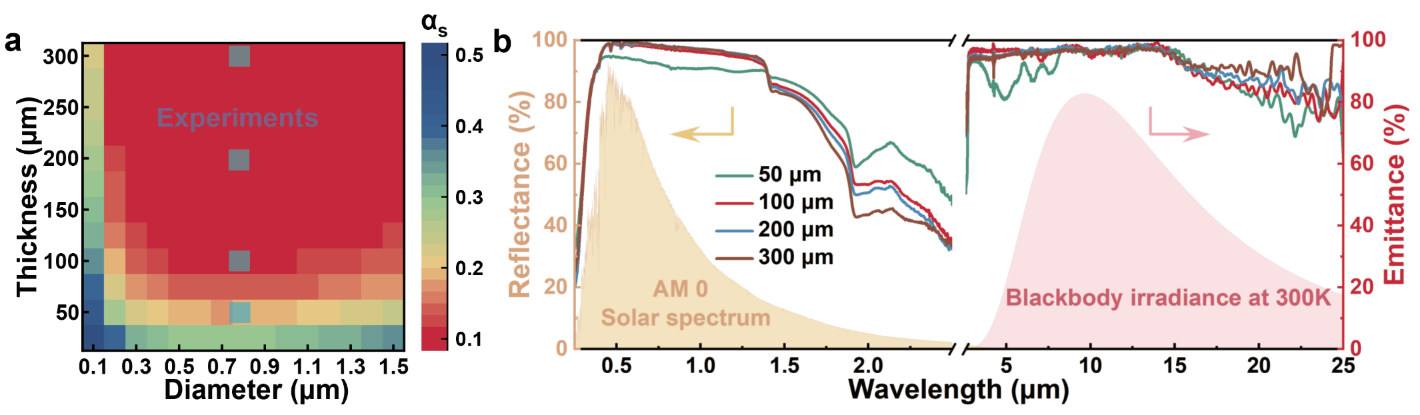
**

**Fig. S18 a** Grid search mapping of the optimal *α_s_* region for metacoatings at 35% volume fraction as a function of diameter and thickness. **b** Reflectance and emittance spectra for metacoatings with varying thicknesses

**Table S1** Comparison of *α_s_*, *ε* and net cooling power of the all-inorganic space radiative cooling coatings

| Materials | *α_s_* | *ε* | Net cooling power (W·m^-2^) | References |
| --- | --- | --- | --- | --- |
| Zn_2_SiO_4_ | 0.091 | 0.949 | 307.32 | [S9] |
| ZnO | 0.167 | 0.88 | 175.87 | [S9] |
| *h*-BN | 0.098 | 0.916 | 286.73 | [S9] |
| Ga_2_O_3_ | 0.094 | 0.877 | 274.28 | [S10] |
| Ca_3_(PO_4_)_2_ | 0.095 | 0.878 | 273.37 | [S10] |
| Zn-MCM-41 | 0.154 | 0.914 | 209.26 | [S11] |
| Silica-supported ZnO | 0.145 | 0.945 | 235.80 | [S12] |
| Zn-SBA-15 | 0.151 | 0.936 | 223.46 | [S13] |
| Hollow glass | 0.16 | 0.85 | 171.66 | [S14] |
| ZrO_2_ particles | 0.205 | 0.84 | 105.55 | [S15] |
| Mg_0.13_Zr_0.87_O_1.87_ | 0.405 | 0.873 | -152.69 | [S16] |
| Mg_3_(PO)_4_ and MgO | 0.33 | 0.85 | -60.73 | [S17] |
| SR107-ZK | 0.17 | 0.87 | 167.18 | [S18] |
| S781 | 0.18 | 0.87 | 153.51 | [S19] |
| NS-74 | 0.17 | 0.92 | 190.14 | [S20] |
| TSSZ-2 | 0.145 | 0.945 | 235.80 | [S12] |
| SAR-9 | 0.09 | 0.80 | 244.39 | [S21] |
| S13G | 0.22 | 0.90 | 112.60 | [S11] |
| YB-71 | 0.12 | 0.90 | 253.79 | [S13] |
| AZ-93 | 0.15 | 0.91 | 212.89 | https://www.aztechnology.com |
| AZW/LA-II | 0.09 | 0.91 | 294.91 |  |
| AZ-2000-IECW | 0.25 | 0.88 | 62.41 |  |
| AZ-2100-IECW | 0.15 | 0.90 | 208.29 |  |
| AZ-400-LSW | 0.17 | 0.89 | 177.73 |  |
| AZJ-4020 | 0.15 | 0.88 | 199.11 |  |
| SolarWhite | 0.18 | 0.89 | 162.69 | https://enbio.eu/thermal-control-coatings/ |
| EZS metacoating | 0.076 | 0.931 | 323.688 | Our work |


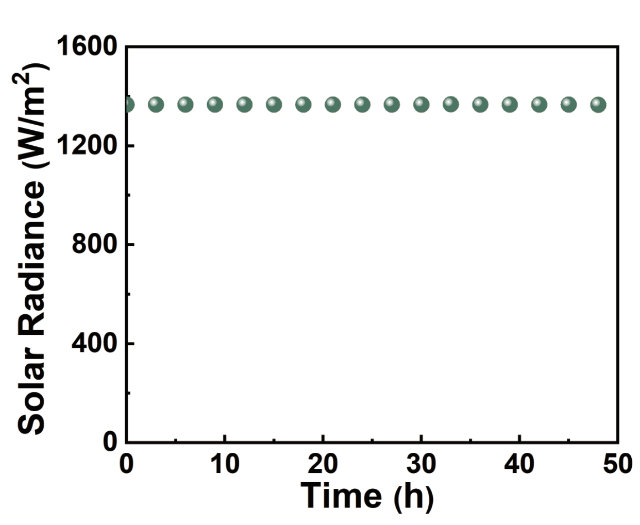


**Fig. S19** Solar irradiance of the AM0 solar simulator as a function of time


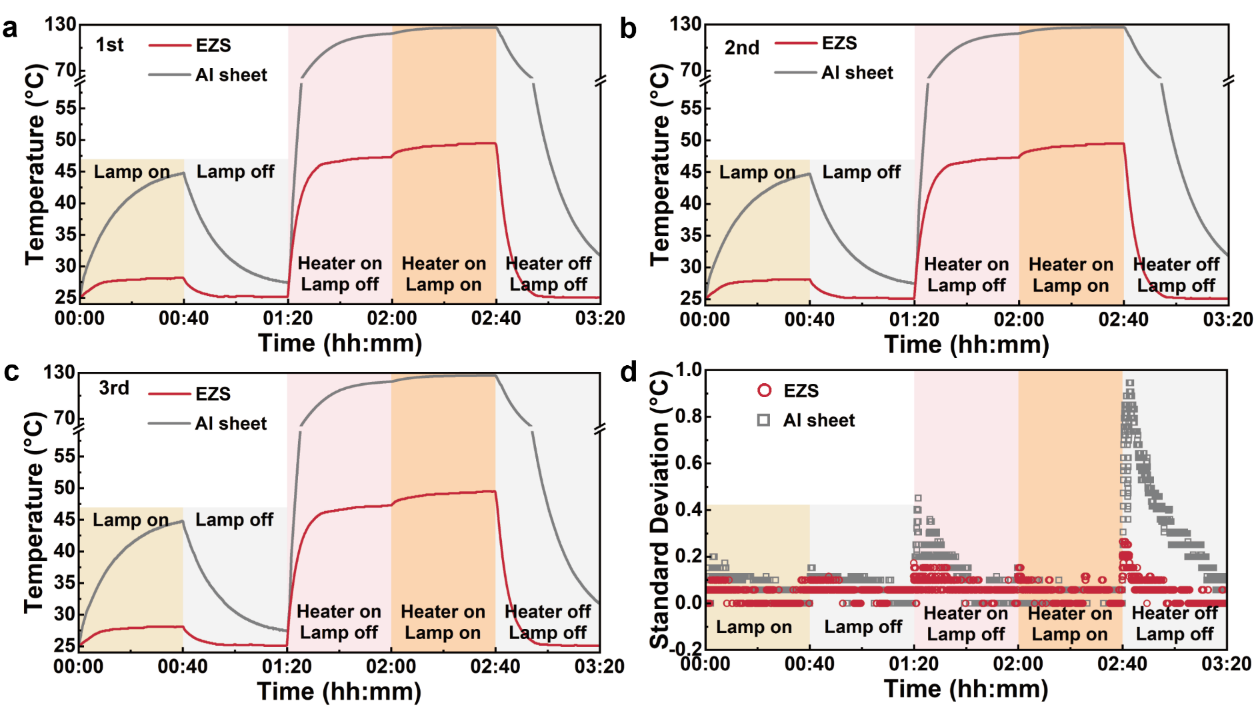


**Fig. S20** Temperature evolution of the EZS metacoating and Al sheet during three independent measurements: **a** first, **b** second, **c** third measurement and **d** corresponding standard deviation


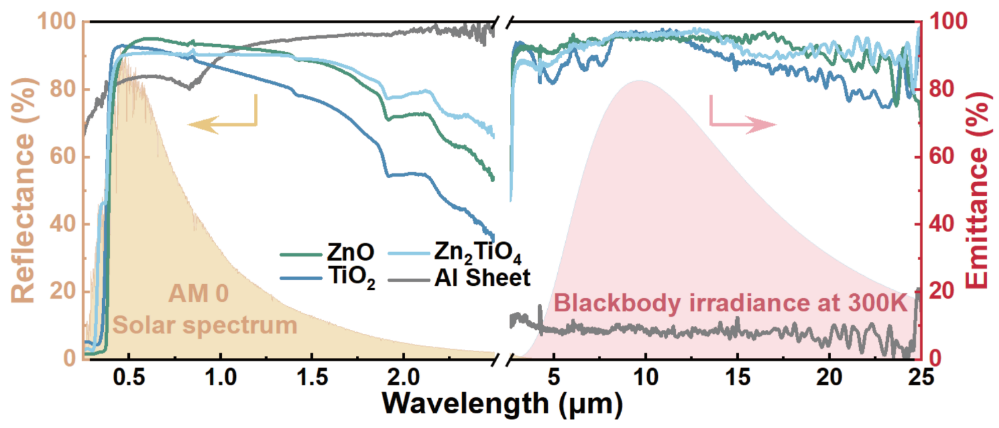


**Fig. S21** The reflectance and emittance spectra of TiO_2_, ZnO and Zn_2_TiO_4_ coatings with thickness of 100 μm at a volume fraction of 35%


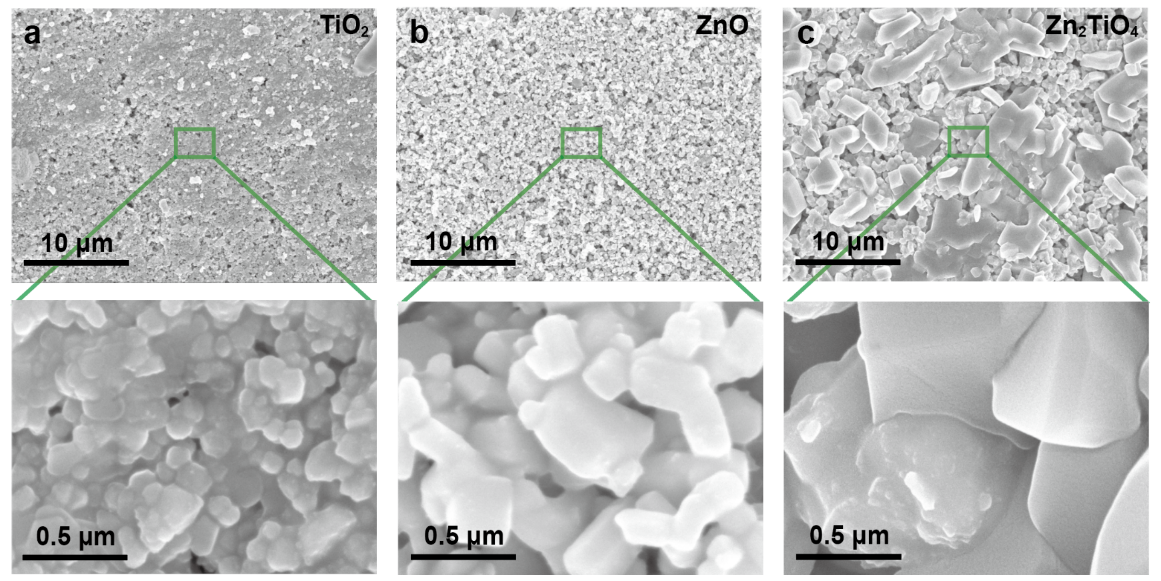


**Fig. S22** The low and high-magnification SEM images of coatings using different oxide powders as pigments: **a** TiO_2_, **b** ZnO and **c** Zn_2_TiO_4_

Bare Al sheet exhibits pronounced intrinsic absorption in the UV-visible range (0.25-0.78 µm), yielding *α_s_* = 0.140 and *ε* = 0.081. Sprayed ZnO, TiO_2_ and Zn_2_TiO_4_ powders form uniform, well-adhered white coatings [S8]. In reflectance, the UV absorption edges of ZnO, TiO_2_ and Zn_2_TiO_4_ coatings occur at ~0.38, ~0.36, and ~0.32 µm, respectively, which leads to stronger UV absorption. SEM images at low and high magnification reveal granular surfaces. TiO_2_ coating consists of smaller particles (~0.1-0.3 µm), which maximize reflectance in the visible region but lead to reduced reflectance in the NIR range. ZnO and Zn_2_TiO_4_ coatings have larger particles and maintain higher NIR reflectance. Because all coatings were prepared at the same pigment volume fraction, their mid-IR emittance spectra are broadly similar. Residual differences are concentrated in the 3-8 µm and 15-25 µm bands and likely arise from intrinsic vibrational absorptions of the oxides and particle-size-dependent interfacial scattering.

**
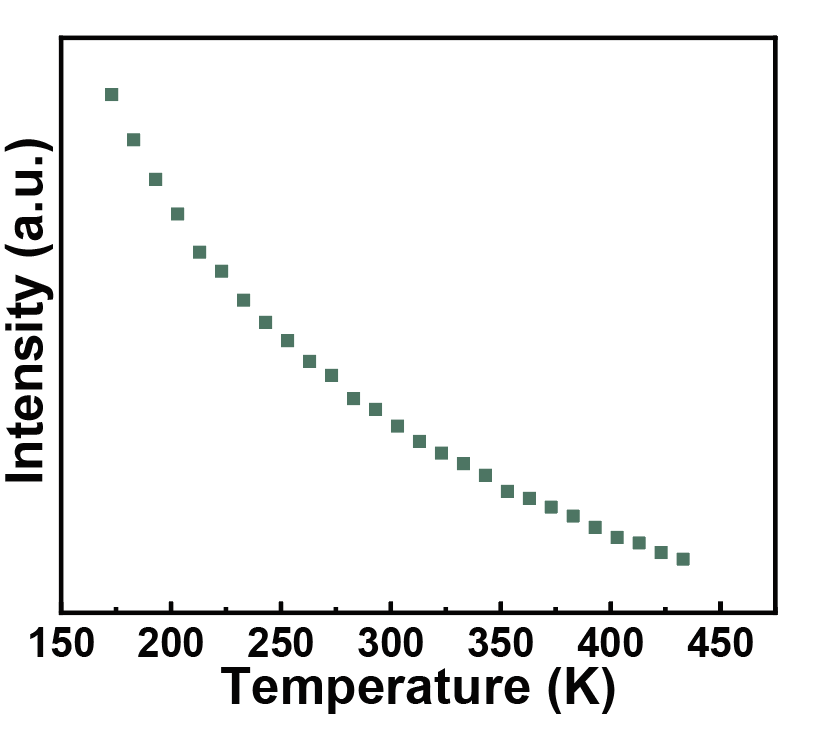
**

**Fig. S23** Variation of intensity of 606 nm with temperature

**Table S2** Comparison of *λ_g_* and maximum *S_r_* for the EZS metacoating and representative luminescent thermometric oxide

| Materials | *λ_g_* | *S_r_* (% K^-1^) | References |
| --- | --- | --- | --- |
| ZrO_2_: Eu^3+^ | 0.258 | 0.33 | [S1] |
| LaScO_3_: Eu^3+^ | 0.281 | 0.795 | [S22] |
| Sr_2_NaMg_2_V_3_O_12_: Eu^3+^ | 0.4133 | 1.61 | [S23] |
| ZrTiO_4_: Eu^3+^ | 0.340 | 1.11 | [S4] |
| Ca_0.75_Sr_0.2_Mg_1.05_Si_2_O_6_: Eu^2+^/Tb^3+^ | 0.448 | 0.6 | [S24] |
| La_2.95_Li_3_W_1.99_O_12_: Eu^3+^/Mn^4+^ | 0.320 | 0.7 | [S25] |
| Gd_2_GaSbO_7_: Bi^3+^/Eu^3+^ | 0.293 | 1.03 | [S26] |
| SrGa_2_B_2_O_7_: Bi^3+^/Eu^3+^ | 0.276 | 1.03 | [S27] |
| CaLa_4_(SiO_4_)_3_O: Dy^3+^ | 0.339 | 0.164 | [S28] |
| SrWO_4_: Pr^3+^ | 0.272 | 0.65 | [S29] |
| BaLa_0.92_La_0.08_LiTeO_6_ | 0.343 | 0.65 | [S30] |
| LiCa_2_Mg_2_V_3_O_12_: Dy^3+^ | 0.397 | 0.59 | [S31] |
| RbCaLa(VO_4_)_2_: Eu^3+^ | 0.362 | 1.125 | [S32] |
| La_2_LiSbO_6_: Bi^3+^/Eu^3+^ | 0.388 | 0.41 | [S33] |
| K_3_Y_4_Si_2_O_7_: Bi^3+^/Sm^3+^ | 0.292 | 0.673 | [S34] |
| Ba_2_LuNbO_6_: Er^3+^/Yb^3+^ | 0.334 | 1.81 | [S35] |
| Sr_3_WO_6_: Er^3+^ | 0.342 | 0.56 | [S36] |
| Gd_2_Mo_3_O_12_: Er^3+^/Yb^3+^ | 0.343 | 0.89 | [S37] |
| NaLaCaWO_6_: Bi^3+^/Eu^3+^ | 0.423 | 0.74 | [S38] |
| LiAl_5_O_8_: Cr^3+^ | 0.225 | 0.3 | [S39] |
| Ca_3_Al_2_O_6_: Bi^3+^/Eu^3+^ | 0.299 | 0.3 | [S40] |
| Ba_2_SrWO_6_: Eu^3+^ | 0.332 | 0.33 | [S41] |
| Ba_2_Al_2/3_TeO_6_: Eu^3+^ | 0.253 | 0.19 | [S42] |
| LiY_6_(BO_3_)_3_O_5_: Bi^3+^/Eu^3+^ | 0.426 | 1.87 | [S43] |
| Y_3_TaO_7_: Tm^3+^/Eu^3+^ | 0.251 | 0.541 | [S44] |
| EZS metacoating | 0.253 | 0.797 | Our work |


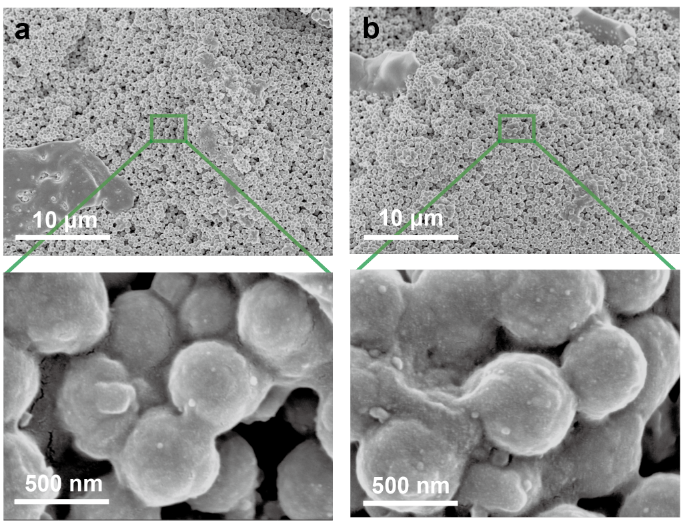


**Fig. S24** The low and high-magnification SEM images of EZS metacoating after **a** UV and **b** combined irradiations

**
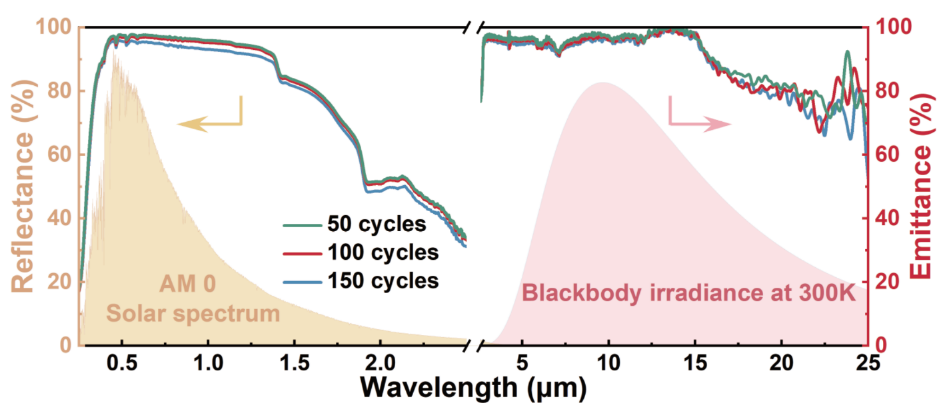
**

**Fig. S25** Reflectance and emittance spectra of the EZS metacoating after 50, 100 and 150 thermal cycles between -196 °C and 150 °C


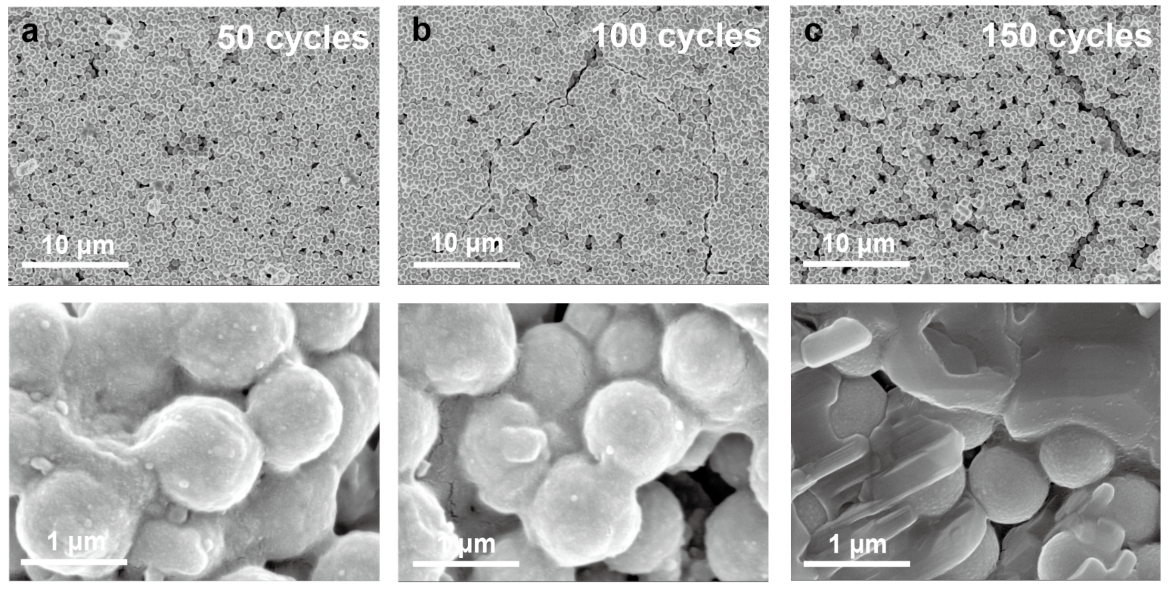


**Fig. S26** The SEM images of EZS metacoating after thermal cycling between -196 °C and 150 °C: **a** 50 cycles, **b** 100 cycles and **c** 150 cycles


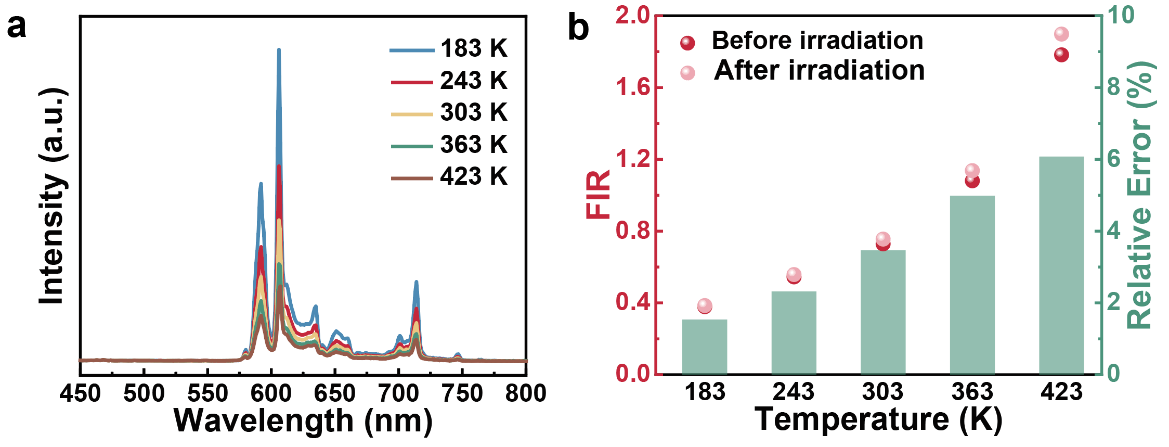


**Fig. S27** **a** Temperature-dependent emission spectra (λ_ex_ = 395 nm) of the EZS metacoating after combined irradiations. **b** FIR values before and after combined irradiation at different temperatures and the corresponding relative error

**Table S3** Comparison of *α_s_* of the space radiative cooling coatings before and after different irradiations

| Materials | Initial *α_s_* | Irradiation type | Final *α_s_* | References |
| --- | --- | --- | --- | --- |
| Zn_2_SiO_4_ | 0.091 | Proton | 0.193 | [S9] |
| ZnO | 0.167 |  | 0.253 | [S9] |
| *h*-BN | 0.098 |  | 0.241 | [S9] |
| NaZnF_3_/SiO_2_ | 0.064 |  | 0.193 | [S45] |
| Ga_2_O_3_ | 0.085 |  | 0.174 | [S10] |
| Ca_3_(PO_4_)_2_ | 0.093 |  | 0.143 | [S10] |
| ZnTiO_3_ | 0.101 |  | 0.521 | [S46] |
| SrTiO_3_ | 0.125 |  | 0.386 | [S46] |
| Z93C55 | 0.15 |  | 0.16 | [S47] |
| EZS | 0.076 |  | 0.090 | Our work |
| AZ-93 | 0.138 | Electron | 0.301 | [S48] |
| AZ-2100 | 0.272 |  | 0.336 | [S48] |
| AZ-2170 | 0.195 |  | 0.3 | [S48] |
| AZW/LA-II | 0.096 |  | 0.273 | [S48] |
| AZO/Al_2_O_3_-ZnO-Y_2_O_3_ | 0.409 |  | 0.426 | [S49] |
| ZnO | 0.138 |  | 0.192 | [S50] |
| BaSO_4_ | 0.088 |  | 0.144 | [S51] |
| TiO_2_ | 0.144 |  | 0.263 | [S51] |
| ZrO_2_ | 0.081 |  | 0.171 | [S52] |
| Z93C55 | 0.15 |  | 0.36 | [S47] |
| EZS | 0.076 |  | 0.110 | Our work |
| AZW/LA-II | 0.096 | AO | 0.100 | [S53] |
| Z-93 | 0.12 |  | 0.15 | [S54] |
| S13G/LO-1 | 0.13 |  | 0.17 | [S54] |
| YB-71 | 0.08 |  | 0.14 | [S54] |
| EZS | 0.076 |  | 0.088 | Our work |
| PEO treated ZrO_2_ | 0.205 | UV | 0.223 | [S15] |
| SolarWhite | 0.18 |  | 0.24 | [S55] |
| SG121FD | 0.245 |  | 0.452 | [S56] |
| Z-93 | 0.15 |  | 0.18 | [S57] |
| YB-71 | 0.10 |  | 0.19 | [S54] |
| EZS | 0.076 |  | 0.159 | Our work |
| YB-71 | 0.090 | Electron and UV | 0.220 | [S58] |
| Z-93 | 0.136 | Electron and UV | 0.225 | [S57] |
| S13G/LO-1 | 0.165 | Electron and UV | 0.306 | [S57] |
| SG121FD | 0.245 | Electron and proton | 0.375 | [S56] |
| S781 | 0.193 | Proton, electron and UV | 0.392 | [S18] |
| SR107-ZK | 0.160 | Proton, electron and UV | 0.604 | [S18] |
| EZS | 0.076 | Proton, electron, AO and UV | 0.174 | Our work |


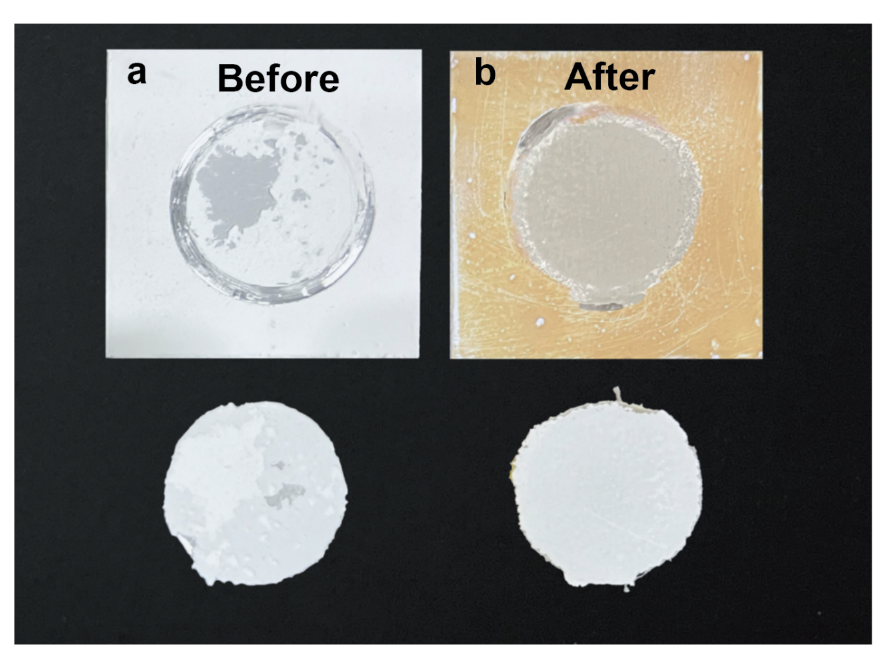


**Fig. S28** Photographs of the EZS metacoating following pull-off testing: **a** before and **b** after combined irradiations


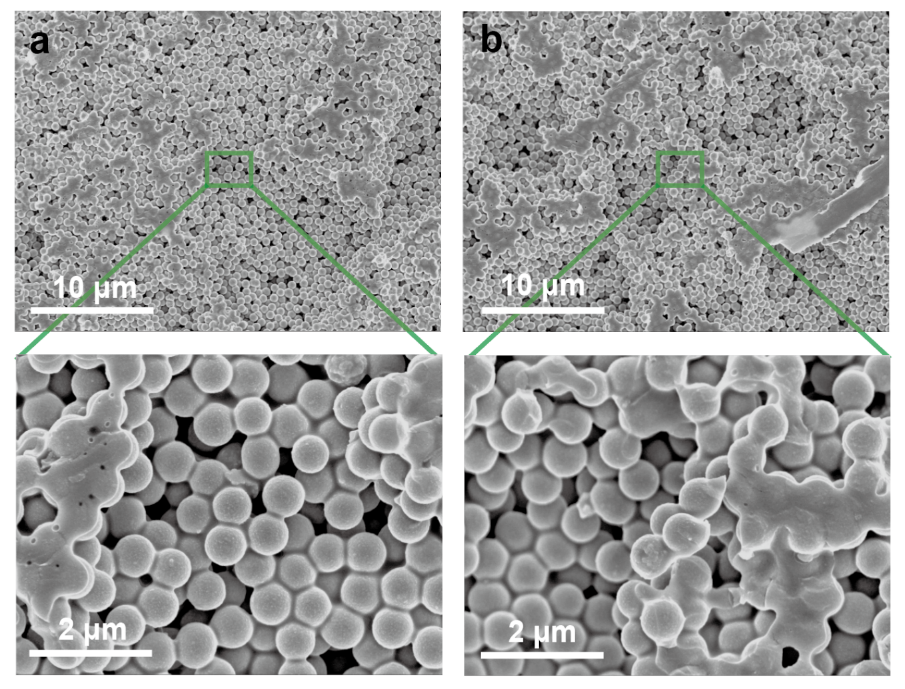


**Fig. S29** The low and high-magnification SEM images of the interface between the EZS metacoating and the Al sheet **a** before and **b** after combined irradiations

**
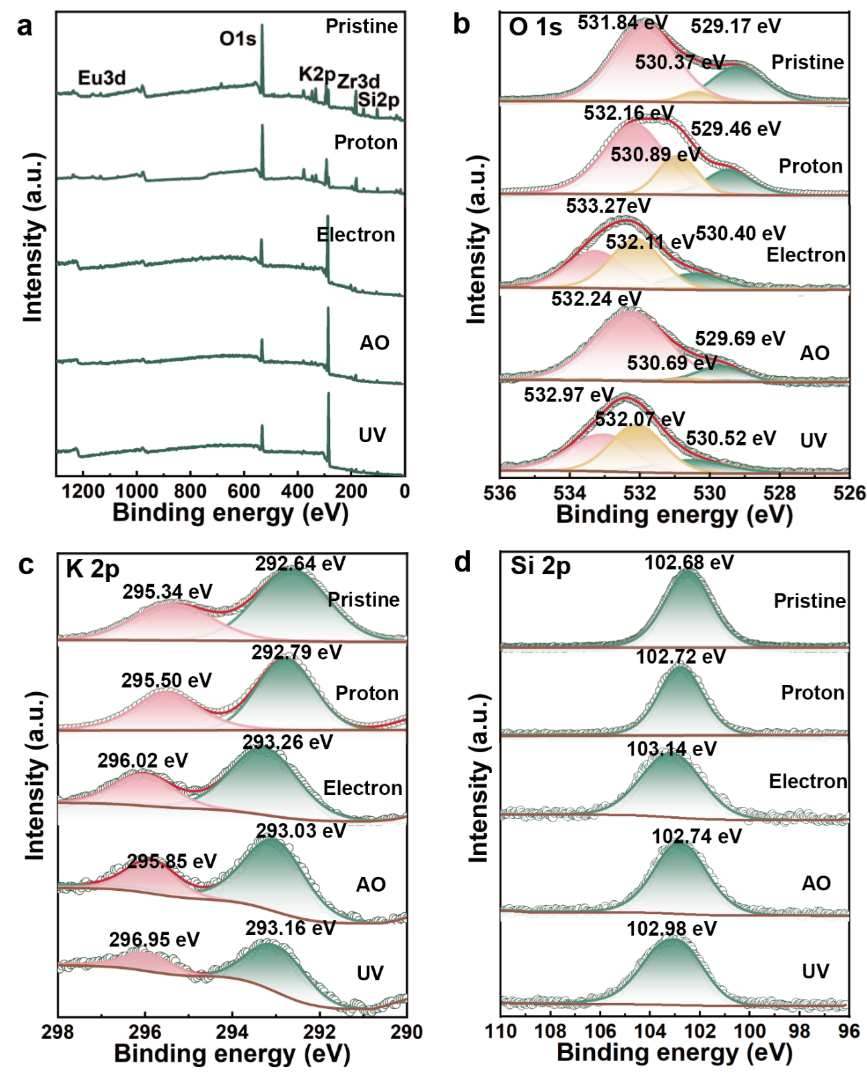
**

**Fig. S30** XPS spectra of EZS metacoating before and after various irradiations: **a** survey spectra, **b** O 1s, **c** K 2p and **d** Si 2p high-resolution spectra

As shown in Fig. S30a, the XPS survey spectra confirm that the EZS metacoating primarily consists of Zr, O, Eu, K and Si. The high-resolution O 1s spectrum (Fig. S30b) of the pristine metacoating shows components at 529.17 eV (lattice oxygen, O_L_), 530.37 eV (hydroxyl, −OH) and 531.84 eV (Si−O). After proton and AO irradiations, a slight binding-energy shift toward higher values occurs. The −OH increases under proton exposure but decreases after AO irradiation, reflecting their distinct redox mechanisms: proton irradiation introduces hydrogen-related defects, whereas AO exposure promotes surface oxidation. Electron and UV irradiations cause more significant changes, shifting the O 1s peaks to 530.40 eV, 532.11 eV and 533.27 eV (electron) and to 530.52 eV, 532.07 eV and 532.97 eV (UV), accompanied by enhanced −OH intensity. These shifts arise from charge accumulation induced by high-energy electron bombardment or photoexcitation, which increases surface potential and promotes hydroxyl enrichment.

The K 2p spectra (Fig. S30c) further elucidate the chemical state of potassium. In the pristine metacoating, the K 2p_3/2_ and K 2p_1/2_ peaks appear at 292.64 eV and 295.34 eV with a spin-orbit splitting of 2.7 eV, characteristic of monovalent K^+^. Proton and AO irradiations have negligible effects, while electron and UV exposures shift the peaks to 293.26/296.02 eV and 293.16/296.95 eV, respectively, with slightly increased splitting (2.76/2.79 eV). These shifts suggest enhanced localization of the K electron shell, likely due to local charge redistribution.

As shown in Fig. S30d, the Si 2p peak of the pristine metacoating is centered at 102.68 eV, corresponding to Si−O bonding. Proton and AO irradiations induce only minor changes, whereas electron and UV exposures shift the peak to 103.14 eV and 102.98 eV, accompanied by peak broadening. This behavior indicates partial bond-length variations and nonlocal charge rearrangement within the Si−O network under irradiations, reflecting perturbations of the local chemical environment.

**
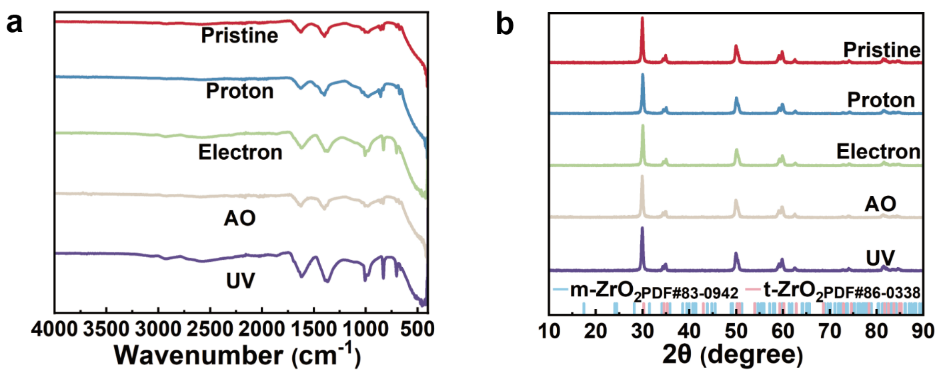
**

**Fig. S31 a** FTIR spectra and **b** XRD patterns of EZS metacoating after proton, electron, AO and UV irradiations

FTIR and XRD were employed to characterize the EZS metacoating after proton, electron, AO, and UV irradiations. As shown in Fig. S31a, the FTIR spectra display no discernible changes after proton or AO exposures. In contrast, electron and UV irradiations markedly increase the bands at 1629 cm^-1^ and 1398 cm^-1^, which are assigned to surface Si−OH vibrations or adsorbed H_2_O and to carbonate (CO_3_^2-^) or physiosorbed CO_2_, respectively [S59–S61]. This enhancement suggests that high-energy electrons and UV irradiations generate surface defects that promote physisorption. XRD analysis (Fig. S31b) shows that the metacoatings after different irradiations retain the characteristic tetragonal ZrO_2_ pattern with no peak shifts or emergent reflections, indicating the absence of lattice distortion, phase transformation, or amorphization. Peak intensities and full widths at half maximum also remain essentially unchanged, implying minimal variation in crystallite size and overall crystallinity.

**Supplementary References**

1. J. Zhou, R. Lei, H. Wang, Y. Hua, D. Li et al., A new generation of dual-mode optical thermometry based on ZrO_2_: Eu^3+^ nanocrystals. Nanophotonics **8**(12), 2347–2358 (2018). https://doi.org/10.1515/nanoph-2019-0359
2. J. Kaszewski, B.S. Witkowski, Wachnicki, T. Płociński, L.-I. Bulyk et al., Role of Zr^3+^ in excitation of Eu^3+^ ions in stabilized ZrO_2_: Eu nanoparticles. J. Lumin. **273**, 120654 (2024). https://doi.org/10.1016/j.jlumin.2024.120654
3. X. Zhang, Y. Xu, X. Wu, S. Yin, C. Zhong et al., Optical thermometry and multi-mode anti-counterfeiting based on Bi^3+^/Ln^3+^ and Ln^3+^ doped Ca_2_ScSbO_6_ phosphors. Chem. Eng. J. **481**, 148717 (2024). https://doi.org/10.1016/j.cej.2024.148717
4. A. Gu, G.-H. Pan, H. Wu, L. Zhang, L. Zhang et al., Microstructure and photoluminescence of ZrTiO_4_: Eu^3+^ phosphors: host-sensitized energy transfer and optical thermometry. Chemosensors **10**(12), 527 (2022). https://doi.org/10.3390/chemosensors10120527
5. Y. Xia, C. Zhang, J.-X. Wang, D. Wang, X.-F. Zeng et al., Synthesis of transparent aqueous ZrO_2_ nanodispersion with a controllable crystalline phase without modification for a high-refractive-index nanocomposite film. Langmuir **34**(23), 6806–6813 (2018). https://doi.org/10.1021/acs.langmuir.8b00160
6. M.T. Colomer, Straightforward synthesis of Ti-doped YSZ gels by chemical modification of the precursors alkoxides. J. Sol Gel Sci. Technol. **67**(1), 135–144 (2013). https://doi.org/10.1007/s10971-013-3059-9
7. M. Tahmasebpour, A.A. Babaluo, M.K.R. Aghjeh, Synthesis of zirconia nanopowders from various zirconium salts *via* polyacrylamide gel method. J. Eur. Ceram. Soc. **28**(4), 773–778 (2008). https://doi.org/10.1016/j.jeurceramsoc.2007.09.018
8. H. Gong, L. Tong, Z. Wang, X. Song, H. Li et al., Material and structure tailored La-doped ZrO_2_ submicrosphere metacoatings for high-performance space radiative cooling. Adv. Funct. Mater. e28343 (2026). https://doi.org/10.1002/adfm.202528343
9. J. Lv, J. Xie, N. Vitaly, Development of Zn_2_SiO_4_ and hexagonal BN inorganic thermal-control coatings with novel thermophysical property. Int. J. Heat Mass Transf. **218**, 124791 (2024). https://doi.org/10.1016/j.ijheatmasstransfer.2023.124791
10. J. Lv, X. Wen, Development of Ca_3_(PO_4_)_2_ inorganic thermal-control coating used in harsh space environments. Sol. Energy Mater. Sol. Cells **237**, 111578 (2022). https://doi.org/10.1016/j.solmat.2022.111578
11. N. Kiomarsipour, R. Shoja Razavi, K. Ghani, Improvement of spacecraft white thermal control coatings using the new synthesized Zn-MCM-41 pigment. Dyes Pigm. **96**(2), 403–406 (2013). https://doi.org/10.1016/j.dyepig.2012.08.019
12. V. Heydari, Z. Bahreini, Synthesis of silica-supported ZnO pigments for thermal control coatings and analysis of their reflection model. J. Coat. Technol. Res. **15**(1), 223–230 (2018). https://doi.org/10.1007/s11998-017-9969-7
13. V. Heydari, Z. Bahreini, M. Heidari, A. Sedrpoushan, Synthesis of Zn-SBA-15 as a new pigment for spacecraft white thermal control coatings. J. Coat. Technol. Res. **13**(4), 727–733 (2016). https://doi.org/10.1007/s11998-015-9778-9
14. Y. Yang, M. Jiang, Z. Ma, A.A. Rogachev, W. Tian et al., The coating with ZrO_2_-coated hollow glass microspheres: Low solar absorption and high microwave transmittance. Mater. Des. **232**, 112136 (2023). https://doi.org/10.1016/j.matdes.2023.112136
15. J. Liang, Z. Peng, R. Li, B. Wang, Preparation of white ZrO_2_ coating with low solar absorptance on aluminum alloy by plasma electrolytic oxidation. Ceram. Int. **49**(17), 29133–29140 (2023). https://doi.org/10.1016/j.ceramint.2023.06.192
16. H. Li, S. Lu, X. Wu, W. Qin, Influence of Zr^4+^ ions on solar absorbance and emissivity of coatings formed on AZ31 Mg alloy by plasma electrolytic oxidation. Surf. Coat. Technol. **269**, 220–227 (2015). https://doi.org/10.1016/j.surfcoat.2015.01.070
17. Z. Yao, P. Ju, Q. Xia, J. Wang, P. Su et al., Preparation of thermal control coatings on Mg–Li alloys by plasma electrolytic oxidation. Surf. Coat. Technol. **307**, 1236–1240 (2016). https://doi.org/10.1016/j.surfcoat.2016.06.019
18. W. Feng, Y. Ding, D. Yan, X. Liu, W. Wang et al., Combined low-energy environment stimulation test of geosynchronous satellite thermal control coatings. J. Spacecr. Rockets **46**(1), 11–14 (2009). https://doi.org/10.2514/1.26291
19. [S19] W.Q. Feng, Y.G. Ding, D.K. Yan. Space combined environment simulation test on α_s_ degradation of GEO satellite thermal control coatings. Spacecraft Environ. Eng. 24, 27 (2007). https://seejournal.cn/cn/article/pdf/preview/226c2a2f-ed28-497a-8ada-6e8d40e08c44
20. V. Alcayde, A. Vercher-Martínez, F.J. Fuenmayor, Thermal control of a spacecraft: Backward-implicit scheme programming and coating materials analysis. Adv. Space Res. **68**(4), 1975–1988 (2021). https://doi.org/10.1016/j.asr.2021.03.041
21. W. Qin, X.H. Wu, G.M. Zhao, X.M. Lai, L.G. Zhang, Optical absorption properties of nanotitanium dioxide doped ZnO/silicone thermal control coating. Mater. Sci. Forum **546–549**, 1725–1728 (2007). https://doi.org/10.4028/www.scientific.net/msf.546-549.1725
22. D. Huang, Y. Wei, P. Dang, X. Xiao, H. Lian et al., Tunable color emission in LaScO_3_: Bi^3+^, Tb^3+^, Eu^3+^ phosphor. J. Am. Ceram. Soc. **103**(5), 3273–3285 (2020). https://doi.org/10.1111/jace.17007
23. A. Bindhu, J.I. Naseemabeevi, S. Ganesanpotti, Vibrationally induced photophysical response of Sr_2_NaMg_2_V_3_O_12_: Eu^3+^ for dual-mode temperature sensing and safety signs. Adv. Photonics Res. **3**(6), 2100159 (2022). https://doi.org/10.1002/adpr.202100159
24. K. Su, Q. Guo, P. Shuai, N. Liu, L. Mei et al., A novel Eu^2+^/Tb^3+^ Co-doped phosphor with pyroxene structure applied for cryogenic thermometric sensing. J. Am. Ceram. Soc. **105**(4), 2903–2913 (2022). https://doi.org/10.1111/jace.18275
25. Q. Tang, N. Guo, Y. Xin, W. Li, B. Shao et al., Luminous tuning in Eu^3+^/Mn^4+^ Co-doped double perovskite structure by designing the site-occupancy strategy for solid-state lighting and optical temperature sensing. Mater. Res. Bull. **149**, 111704 (2022). https://doi.org/10.1016/j.materresbull.2021.111704
26. L. Ma, F. Lu, Q. Yu, P. Dai, F. Hu et al., A three-mode optical thermometry based on thermochromic Gd_2_GaSbO_7_: Bi^3+^, Eu^3+^ phosphors. Ceram. Int. **49**(11), 16681–16689 (2023). https://doi.org/10.1016/j.ceramint.2023.02.029
27. X. Liu, S. Shi, K. Yang, L. Chen, D. Deng et al., Temperature sensing characteristics of Bi^3+^/Eu^3+^ co-activated SrGa_2_B_2_O_7_: Phosphor for dual-mode optical thermometry. J. Alloys Compd. **879**, 160247 (2021). https://doi.org/10.1016/j.jallcom.2021.160247
28. Q. Liu, M. Wu, B. Chen, X. Huang, M. Liu et al., Optical thermometry based on fluorescence intensity ratio of Dy^3+^-Doped oxysilicate apatite warm white phosphor. Ceram. Int. **49**(3), 4971–4978 (2023). https://doi.org/10.1016/j.ceramint.2022.10.012
29. J. Zheng, H. Shen, Y. Li, H. Li, Z. Yue, Structural and luminescent performance and optical thermometry of Pr^3+^ doped SrWO_4_ down-conversion phosphors. J. Alloys Compd. **968**, 172112 (2023). https://doi.org/10.1016/j.jallcom.2023.172112
30. A.K. Sreelekshmi, S.P. Shamsudeen, S.D. Rani, D.N. Bhadran, R.G. Abhilash Kumar, Unraveling the photophysical response of BaLaLiTeO_6_: Dy^3+^ double perovskites with excellent thermal stability for colour tunable LEDs and optical thermometry. Ceram. Int. **50**(23), 50291–50308 (2024). https://doi.org/10.1016/j.ceramint.2024.09.374
31. M. Fu, Z. Fan, P. Qiao, L. Hu, S. Liu et al., Dual-mode optical thermometers *via* the thermochromic Bi^3+^, Eu^3+^ Co-doped La_2_LiSbO_6_ phosphors for real-time chip temperature monitoring. Ceram. Int. **50**(15), 26454–26463 (2024). https://doi.org/10.1016/j.ceramint.2024.04.372
32. X. Huang, C. He, X. Zhu, C. Yang, Y. Liu et al., Synthesis and characterization of Eu^3+^-doped RbCaLa(VO_4_)_2_ phosphors and influence of temperature on fluorescence properties. Ceram. Int. **47**(22), 32130–32137 (2021). https://doi.org/10.1016/j.ceramint.2021.08.104
33. R. Raji, P.S. Anjana, N. Gopakumar, An insight into Judd-Ofelt analysis and non-contact optical thermometry of LiCa_2_Mg_2_V_3_O_1_: Dy^3+^ phosphors for multifunctional applications. Opt. Mater. **145**, 114393 (2023). https://doi.org/10.1016/j.optmat.2023.114393
34. Q. Wang, S. Zhao, J. Wen, X. Huang, C. Wei et al., A self-referenced fluorescence intensity ratio optical temperature sensing materials K_3_YSi_2_O_7_: Bi^3+^/Sm^3+^based on multi-strategy combination. Ceram. Int. **49**(24), 41264–41271 (2023). https://doi.org/10.1016/j.ceramint.2023.03.320
35. Y. Chen, J. Chen, Y. Luo, Q. Wang, H. Guo, Ba_2_LuNbO_6_: Er^3+^, Yb^3+^ up-conversion phosphors for dual-mode thermometry based on fluorescence intensity ratio. J. Am. Ceram. Soc. **107**(12), 8246–8255 (2024). https://doi.org/10.1111/jace.20058
36. N. Degda, N. Patel, K. Chaudhari, K.V.R. Murthy, M. Srinivas, Ratiometric thermometry using down-conversion luminescence and solid-state lighting application of Er^3+^ activated strontium tungstate phosphor. Phys. B Condens. Matter **683**, 415923 (2024). https://doi.org/10.1016/j.physb.2024.415923
37. H. Gou, Q. Wu, L. Luo, W. Li, P. Du, Reengineering the thermometric behaviors of Er^3+^/Yb^3+^-codoped Gd_2_Mo_3_O_12_ microparticles *via* dual-mode luminescence manipulation. Ceram. Int. **49**(23), 38297–38304 (2023). https://doi.org/10.1016/j.ceramint.2023.09.162
38. X. Xue, S. Yang, W. Liu, L. Dong, Q. Zhu et al., NaLaCaWO_6_ double perovskite: Structure analysis, efficient Bi^3+^ to Eu^3+^ energy transfer and favourable visual optical thermometry. J. Mol. Struct. **1333**, 141797 (2025). https://doi.org/10.1016/j.molstruc.2025.141797
39. Y. Yan, F. Huang, G. Zhu, Y. Zhang, Z. Gao et al., Unique spectral broadening induced by exchange coupling between Cr^3+^ ions in LiAl_5_O_8_: Cr^3+^ phosphors for versatile optical applications. Laser Photonics Rev. **19**(6), 2401588 (2025). https://doi.org/10.1002/lpor.202401588
40. Y. Li, J. Song, L. Meng, M. Jiao, Q. Xu et al., Structure, luminescence property, and optical thermometry behavior of novel Bi^3+^-Eu^3+^ codoped tricalcium aluminate phosphor. J. Alloys Compd. **1010**, 178079 (2025). https://doi.org/10.1016/j.jallcom.2024.178079
41. N. Thakan, R. Lohan, A.S. Rao, N. Deopa, Structural and Luminescence attributes of Eu^3+^ ions activated Ba_2_SrWO_6_ phosphor for non-contact optical thermometry applications. J. Mol. Struct. **1346**, 143169 (2025). https://doi.org/10.1016/j.molstruc.2025.143169
42. G.L. Bhagyalekshmi, S. Ganesanpotti, Comprehension of the photoinduced charge transfer assisted energy transfer in Gd^3+^ based host sensitized tellurate phosphors for thermal sensing and anticounterfeiting labels. Dalton Trans. **53**(19), 8229–8242 (2024). https://doi.org/10.1039/d4dt00259h
43. X. Fan, L. Xu, W. Liu, F. Yin, J. Xu et al., Energy transfer in dual-emission LiY_6_(BO_3_)_3_O_5_: Bi^3+^, Eu^3+^ phosphors for temperature sensing applications. Ceram. Int. **50**(18), 32583–32590 (2024). https://doi.org/10.1016/j.ceramint.2024.06.066
44. Y. Xue, K. Qiang, F. Zhao, H. Yang, Q. Mao et al., Multiple linear regression-enhanced optical thermometry *via* phonon-assisted back energy transfer in Tm^3+^-Eu^3+^ Co-doped phosphors. Adv. Opt. Mater. **13**(25), e01050 (2025). https://doi.org/10.1002/adom.202501050
45. H. Chen, K. Xu, P. Li, J. Ma, Full solar-spectral reflectance and radiation stability of NaZnF_3_ QDs/SiO_2_ composite pigments as thermal control pigment. Ceram. Int. **48**(23), 35432–35437 (2022). https://doi.org/10.1016/j.ceramint.2022.08.145
46. M. Fang, J. Lv, Zn_2_SiO_4_ as an ultralow solar absorptive pigment for thermal control coating. Mater. Lett. **255**, 126538 (2019). https://doi.org/10.1016/j.matlet.2019.126538
47. M. Hasegawa, S. Freese, L. Kauder, J. Triolo, “Space Environmental Evaluation of White Conductive Thermal Control Coatings, ” in *42nd AIAA Thermophysics Conference*, (**2011**): 3948. https://arc.aiaa.org/doi/abs/10.2514/6.2011-3948
48. A. Sokolovskiy, E. Plis, R. Hoffmann, M. Bengtson, D. Ferguson, Study of the optical property degradation of white thermal control coatings under high energy electron irradiation. Surf. Coat. Technol. **451**, 129030 (2022). https://doi.org/10.1016/j.surfcoat.2022.129030
49. T. Li, H. Kang, S. Lu, W. Qin, X. Wu, Preparation of antistatic AZO/Al_2_O_3_-ZnO-Y_2_O_3_ composite thermal control coating and its study on electron-resistance radiation performance. Nucl. Instrum. Meth. Phys. Res. Sect. B Beam Interact. Mater. At. **518**, 1–6 (2022). https://doi.org/10.1016/j.nimb.2022.03.004
50. M.M. Mikhailov, A.N. Lapin, A.N. Sokolovskiy, S.A. Yuryev, Effect of atmosphere on recovery of diffuse reflection spectra of ZnO powder modified with nanoparticles upon irradiation in vacuum. Opt. Mater. **115**, 111038 (2021). https://doi.org/10.1016/j.optmat.2021.111038
51. M.M. Mikhailov, S.A. Yuryev, A.N. Lapin, Prospects for applying BaSO_4_ powders as pigments for spacecraft thermal control coatings. Acta Astronaut. **165**, 191–194 (2019). https://doi.org/10.1016/j.actaastro.2019.09.009
52. M.M. Mikhailov, V.V. Neshchimenko, A.N. Sokolovskiy, V.Y. Yurina, Thermal control coatings based on pigments modified with Al_2_O_3_ nanoparticles. Prog. Org. Coat. **131**, 340–345 (2019). https://doi.org/10.1016/j.porgcoat.2019.03.001
53. [S53] M. Choi, “AZW-LA-II White paint on swift: lessons learned from first time flying on spacecraft radiators,” in *2nd International Energy Conversion Engineering Conference*, (**2004**): 5685. https://arc.aiaa.org/doi/abs/10.2514/6.2004-5685
54. [S54] J. Dever, E. Rodriguez, W. Slemp, J. Stoyack, “Evaluation of thermal control coatings for use on solar dynamic radiators in low earth orbit,” in *26th Thermophysics Conference*, (**1991**): 1327. https://arc.aiaa.org/doi/abs/10.2514/6.1991-1327
55. K.A.J. Doherty, B. Twomey, S. McGlynn, N. MacAuliffe, A. Norman et al., High-temperature solar reflector coating for the solar orbiter. J. Spacecr. Rockets **53**(6), 1077–1084 (2016). https://doi.org/10.2514/1.A33561
56. A.K. Sharma, N. Sridhara, Degradation of thermal control materials under a simulated radiative space environment. Adv. Space Res. **50**(10), 1411–1424 (2012). https://doi.org/10.1016/j.asr.2012.07.010
57. C.A. Cerbus, P.S. Carlin, Evaluation of reformulated thermal control coatings in a simulated space environment. Part 2. S-13GP/LO-1 and Z-93P. AIP Conf. Proc. **361**(1), 851–856 (1996). https://doi.org/10.1063/1.49967
58. D.A. Jaworske, S.E. Kline, “*Review of end-of-life thermal control coating performance*.” No. NASA/TM-2008-215173. 2008. https://ntrs.nasa.gov/citations/20080018585
59. Z. Buniazet, J. Couble, S. Maury, A. Cabiac, S. Loridant et al., Acidity of SiO_2_-supported metal oxides in the presence of H_2_O using the AEIR method: 2. adsorption and coadsorption of NH_3_ and H_2_O on TiO_2_/SiO_2_ catalysts. Langmuir **36**(45), 13383–13395 (2020). https://doi.org/10.1021/acs.langmuir.0c01717
60. D.H. Lee, R.A. Condrate, An FTIR spectral investigation of the structural species found on alumina surfaces. Mater. Lett. **23**(4–6), 241–246 (1995). https://doi.org/10.1016/0167-577X(95)00039-9
61. Musyarofah, S. Soontaranon, W. Limphirat, Triwikantoro, S. Pratapa, XRD, WAXS, FTIR, and XANES studies of silica-zirconia systems. Ceram. Int. **45**(12), 15660–15670 (2019). https://doi.org/10.1016/j.ceramint.2019.05.078
